# Reversing VTN deficiency inhibits the progression of pancreatic cancer and enhances sensitivity to anti-PD1 immunotherapy

**DOI:** 10.3389/fimmu.2025.1578870

**Published:** 2025-05-13

**Authors:** Siqi Zhao, Zhaofeng Gao, Lingyu Hu, Yihan Li, Xiaoguang Wang, Xiaoping Li, Minjie Chen, Fei Chen, Zhengwei Song

**Affiliations:** ^1^ Department of Surgery, The Second Affiliated Hospital of Jiaxing University, Jiaxing, Zhejiang, China; ^2^ Department of Surgery, The First Affiliated Hospital of Nanjing Medical University, Nanjing, Jiangsu, China

**Keywords:** vitronectin, pancreatic cancer, tumor progression, immunotherapy, anti-PD1

## Abstract

**Background:**

Pancreatic cancer, a highly lethal malignancy with limited therapeutic options, necessitates the identification of novel prognostic biomarkers and therapeutic targets. The extracellular matrix protein vitronectin (VTN) has been implicated in tumor progression, but its specific role in pancreatic cancer progression and immunotherapy response remains unclear.

**Methods:**

This study employed an integrative approach combining single-cell RNA sequencing, analysis of public databases, and functional assays. *In vitro* experiments assessed the impact of VTN knockdown and overexpression on pancreatic cancer cell proliferation, invasion, and migration. Mechanistic investigations explored associations between VTN expression and immune regulatory factors. A syngeneic mouse subcutaneous tumor model evaluated the therapeutic efficacy of VTN overexpression combined with anti-PD1 immunotherapy.

**Results:**

VTN was significantly downregulated in pancreatic cancer tissues compared to normal tissues. Lower VTN levels correlated with poorer overall survival. VTN knockdown promoted pancreatic cancer cell proliferation, invasion, and migration *in vitro*, whereas VTN overexpression suppressed these phenotypes. VTN expression was linked to immune regulatory pathways. High VTN levels predicted improved survival in patients receiving anti-PD1/PD-L1 therapy. In a mouse model, VTN overexpression inhibited tumor growth and synergized with anti-PD1 therapy to enhance antitumor efficacy, suggesting combinatorial therapeutic potential.

**Conclusions:**

This study identifies VTN as a dual-functional regulator in pancreatic cancer, acting as both a suppressor of tumor progression and a modulator of immunotherapy response. These findings position VTN as a prognostic biomarker and a therapeutic target to sensitize pancreatic tumors to anti-PD1-based immunotherapy, providing a potential strategy for overcoming treatment resistance in this aggressive malignancy.

## Introduction

According to the most recent cancer statistics from 2020, pancreatic cancer, as one of the malignancies with the grimmest prognoses, has an incidence-to-mortality ratio that is alarmingly close to one-to-one. It ranks as the seventh leading cause of cancer-related deaths, with a five-year survival rate of approximately 9 percent (1, 2). A study encompassing 28 European nations has projected that by the year 2025, pancreatic cancer is expected to surpass breast cancer as the third leading cause of cancer-related mortality (3). Risk factors for pancreatic cancer include smoking, alcohol consumption, chronic pancreatitis, diabetes, and family history (4–6). The principal diagnostic modalities for pancreatic cancer presently include radiological imaging (such as CT scans and MRIs), endoscopic ultrasonography (EUS), and biomarkers (like CA19–9 levels in the blood), among others (7).

Depending on the stage of the cancer, treatment may include surgery, chemotherapy, targeted therapy, and/or radiation (8, 9). Surgery is still the most basic and reliable means for the treatment of pancreatic cancer, but due to the occult nature of pancreatic cancer, once discovered, it is advanced, and the postoperative recurrence rate can be as high as 80% within one year (5, 10). The chemotherapeutic regimens for pancreatic cancer typically include agents such as gemcitabine and nab-paclitaxel, and for certain patients with locally advanced or metastatic disease, chemotherapy may represent their sole therapeutic option (11, 12). Radiotherapy is used in some cases to control tumors that are not suitable for surgical removal, or as part of pre-surgery (neoadjuvant therapy) or post-surgery (adjuvant therapy) to reduce the risk of recurrence (13, 14). However, due to the characteristics of pancreatic cancer and the side effects and drug resistance of the above treatment, the treatment of pancreatic cancer is still a long way to go. In recent years, immunotherapy has become a hot spot in the field of tumor therapy. Checkpoint inhibitors such as anti-PD1 and anti-PD-L1 have been shown to be effective against pancreatic cancer alone or in combination with other treatment options (15, 16). In addition, some cancer vaccines, such as GVAX and CRS-207, can recognize and attack cancer cells by activating the immune system (17–19). CAR T cell therapy is also a hot topic at the moment (20). However, akin to patients with other tumors, a considerable proportion of those afflicted with pancreatic cancer exhibit primary or secondary resistance to treatment. This therapeutic impasse has galvanized further investigation into various combination approaches or innovative modalities to augment the efficacy of immunotherapy.

VTN, also known as plasma spreading factor or serum spreading factor, is a glycoprotein ubiquitously present in human plasma and the extracellular matrix (21, 22). As an integral component of the extracellular matrix, VTN plays a pivotal role in the modulation of cell adhesion, migration, proliferation, and apoptosis, as well as in the intricate orchestration of the coagulation and fibrinolytic systems (23–25). VTN contains multiple domains that bind to both surface cells and extracellular matrices and fibers to regulate cell diffusion and migration (26–29). During wound healing, VTN can promote cell migration to the injured area to promote the growth and recovery of new tissue (30, 31). In the regulation of coagulation and fibrolysis system, VTN can be combined with thrombin-antithrombin complex (TAT) to inhibit thrombin formation (32). In addition, it regulates fibrinolysis by binding to plasminogen activator inhibitor-1 (PAI-1), affecting wound healing and tissue remodeling processes (33, 34). Earlier studies also found that VTN binds to the complement C5b-9 complex to suppress excessive immune responses (35). VTN’s role in cancer is concentrated in breast, ovarian, and prostate cancers, and previous studies have shown that it can be used as a serum marker for early cancer detection (36–39). However, its role in pancreatic cancer has not yet been reported. This study reveals that the expression of VTN is diminished in pancreatic cancer and that its expression correlates with patient prognosis and the efficacy of immunotherapy, suggesting that VTN may serve as a potential prognostic biomarker and a sensitizer target for anti-PD1 treatment in pancreatic cancer.

## Method

### Analysis of VTN expression

The resources from TCGAportal(www.tcgaportal.org) and UALCAN(http://ualcan.path.uab.edu/) were harnessed to study variations in VTN expression in non-cancerous tissues near tumors as well as in cancerous samples. Furthermore, these databases helped in contrasting VTN levels in pancreatic cancer patients taking into account their disease stages, drinking habits, pancreatitis status, and tumor grade.

### Cell culture and transfection

All cells were meticulously cultured in DMEM medium (Gibco, USA), enriched with 10% fetal bovine serum (FBS) (NEWZERUM, Newzerum), and maintained at 37°C within a controlled atmosphere of 5% CO2. This milieu was further fortified with penicillin (100 IU/mL) and streptomycin (100 mg/mL) to preempt microbial contamination. We employed custom-synthesized small interfering RNAs (siRNAs) specific to VTN (si-VTN) and a non-targeting control siRNA (si-NC) procured from KeyGEN BioTECH (Jiangsu, China) for gene silencing endeavors. Transfection was adeptly achieved using Lipofectamine 2000 (Invitrogen, USA) within an Opti-MEM medium (Gibco, USA). Post-transfection, at the 48-hour milestone, cells were harvested for downstream experimental interrogation. The precise si-VTN sequences targeted were as follows: 5’-GGGCTTCAACGTGGACAAGAA-3’, 5’-GTGGACAAGAAGTGCCAGTGT-3’, and 5’-GGACAAGAAGTGCCAGTGTGA-3’. In collaboration with KeyGEN BioTECH (Jiangsu, China), a bespoke lentivirus was engineered to robustly overexpress VTN, designed for assiduous *in vitro* and *in vivo* experimentation. Culturing conditions were adjusted to a density of 1×10^5^ cells per well in a 6-well plate, initially nurtured in 2 ml of media for a diurnal cycle, subsequently transitioning to a coalescence of 1 ml of media, an optimum titer of lentivirus, and 40μL of polyburene (Sigma-Aldrich, USA). A subsequent incubation period of 12–16 hours preceded the restoration to a normal growth medium. To scrupulously evaluate the transfection efficiency, we conducted a qRT-PCR assay.

### RNA extraction and quantitative real-time polymerase chain reaction

Adhering to the established protocol, we successfully isolated total RNA employing the Tiangen RNA extraction kit (China). The quantification of gene expression was performed via the Hieff qRT-PCR SYBR Green Master Mix (YEASEN, China). We utilized glyceraldehyde 3-phosphate dehydrogenase (GAPDH) as the reference gene for normalization. The expression data were analyzed using the relative quantification 2−ΔΔCt method. For the amplification of human VTN, we used the following primer sequences: 5’-TGACCAAGAGTCATGCAAGGG-3’ as the forward primer and 5’-ACTCAGCCGTATAGTCTGTGC-3’ as the reverse primer. Similarly, mouse VTN amplification was facilitated with primers: 5’-AGGCCCTTTTTCATACTAGCCC-3’ (forward) and 5’-AAGCTCGTCACACTGACACTT-3’ (reverse).

### Cell proliferation assay

The PANC1 and PATU8988 cells were meticulously partitioned into experimental and control sets and plated separately in 96-well plates. We introduced 1,000 cells to each well, submerged in a 100 μL aliquot of growth medium, and commenced treatment with a 10 μL dose of CCK-8 solution (RiboBio, China). Leveraging a microplate reader, we recorded the optical density at 450 nm at the commencement of the culture, and subsequently at 24, 48, and 72-hour intervals, in strict compliance with the operational protocols of the reader’s manufacturer (Synergy, USA).

### Transwell migration and invasion assays

In our assay configuration, we allocated 200 μl of serum-deprived DMEM medium to the upper compartment, with 700 μl of DMEM replete with 10% fetal bovine serum filling the lower compartment. Each chamber received an inoculation of 20,000 cells. To gauge the invasive prowess of the cells, we armed the Transwell insert (Corning, USA) with a Matrigel concoction (BD Biosciences, USA), a step omitted for migration proficiency assessment. Following a 24-hour culture in standard incubatory conditions, we cleared the upper reservoir of non-invasive cells and aspirated the spent medium. Subsequently, a fixation with 4% paraformaldehyde for a brief decaminute was employed, followed by the employment of crystal violet (China Kagan) for a quarter-hour staining period, and finalized with a PBS cleanse. The cellular specimens were then subjected to microscopic examination and enumeration.

### Clone forming assays

Both unmodified and modified cells were distributed into a 6-well plate maintaining a seeding density of 1,000 cells per well and were then cultured in DMEM enriched with 10% fetal bovine serum. At the conclusion of a 10-day incubation period, the cells underwent methanol fixation and subsequent staining with GIMSA. This preparatory process was followed by imaging and counting of the resultant colonies.

### Survival analysis

The Kaplan-Meier Plotter platform (available at http://kmplot.com/) offers a potent tool for investigating the ties between gene expression (encompassing mRNA, miRNA, protein, and DNA) and survival outcomes across more than 35,000 samples from 21 cancer varieties. We harnessed this database to discern the impact of VTN expression on the prognosis of pancreatic cancer patients. By constructing Kaplan-Meier survival curves, we could juxtapose the survival experiences of different patient sets, and to bolster our analysis, we computed hazard ratios as well as log-rank P-values, all within the 95% confidence intervals.

### Single-cell analysis

To delve into the expression profile of VTN within individual immune cells, we employed the Tumor Immune Single-cell Hub 2 (TISCH2), accessible at http://tisch.comp-genomics.org/home/. Following this, we broadened our investigation to include the use of CancerSEA, found at both http://biocc.hrbmu.edu.cn/CancerSEA/ and http://202.97.205.69/CancerSEA/. CancerSEA provides comprehensive functional status maps for single cells, delineating 14 distinct functional states among 41,900 cells derived from 25 cancer categories (40). This expansive dataset was instrumental in shedding light on the putative roles VTN may play specifically in pancreatic cancer scenarios.

### Immune-related analysis

TISIDB (http://cis.hku.hk/TISIDB/index.php) is the integration of a variety of heterogeneous data types of portal, is applied to the analysis of the interaction of tumor and immune system (41). Here, we used it to analyze the Spearman correlation between VTN expression and immunomodulators.

### Animal models

The animal studies conducted were rigorously evaluated and given the green light by the Jiaxing University Ethics Committee, aligning with the standards and regulations of animal ethics and welfare. Within our study, twenty C57BL/6 male mice were allocated at random into four specific cohorts: OE-NC, OE-VTN, OE-NC+anti-PD1, and OE-VTN+anti-PD1. Employing 2x10^6^ PANC02 cells, we developed a tumorous model in the designated mice via subdermal injections. This procedure was initiated using either the OE-NC or OE-VTN cell lines. Treatment commenced on day 8 post-cell inoculation, with mice in the anti-PD1 groups receiving intraperitoneal injections of anti-PD1 with a dosage of 6.6 mg/kg. The other groups were administered a PBS volume equivalent to that of the anti-PD1 dosage, and treatments were repeated every four days. We rigorously monitored the general health of the mice daily throughout the experiment. Measurement of the tumors’ dimensions was regularly done using vernier calipers to determine the long (a) and short (b) diameters, and tumor volume was calculated using the equation V=ab^2^/2. These assessments were carried out every four days, enabling us to chart the progression of tumor growth over time. By day 20, the experiment reached its conclusion; the mice were humanely sacrificed in order to collect valuable tumor mass data for comprehensive analysis.

### Statistical analysis

GraphPad Prism 9.0 was the chosen software for performing our statistical analyses. Data were reported as mean values with standard deviation. To evaluate differences between two separate sets of data, we employed the two-tailed Student’s t-test. ANOVA was used for assessing variations both within and across multiple groups. For the prognostic analyses, the Kaplan–Meier Plotter was used. Results were considered statistically significant when the P-value fell below 0.05.

## Result

### Single cell level analysis and subcellular localization of VTN in pancreatic cancer

**Figure 1:**
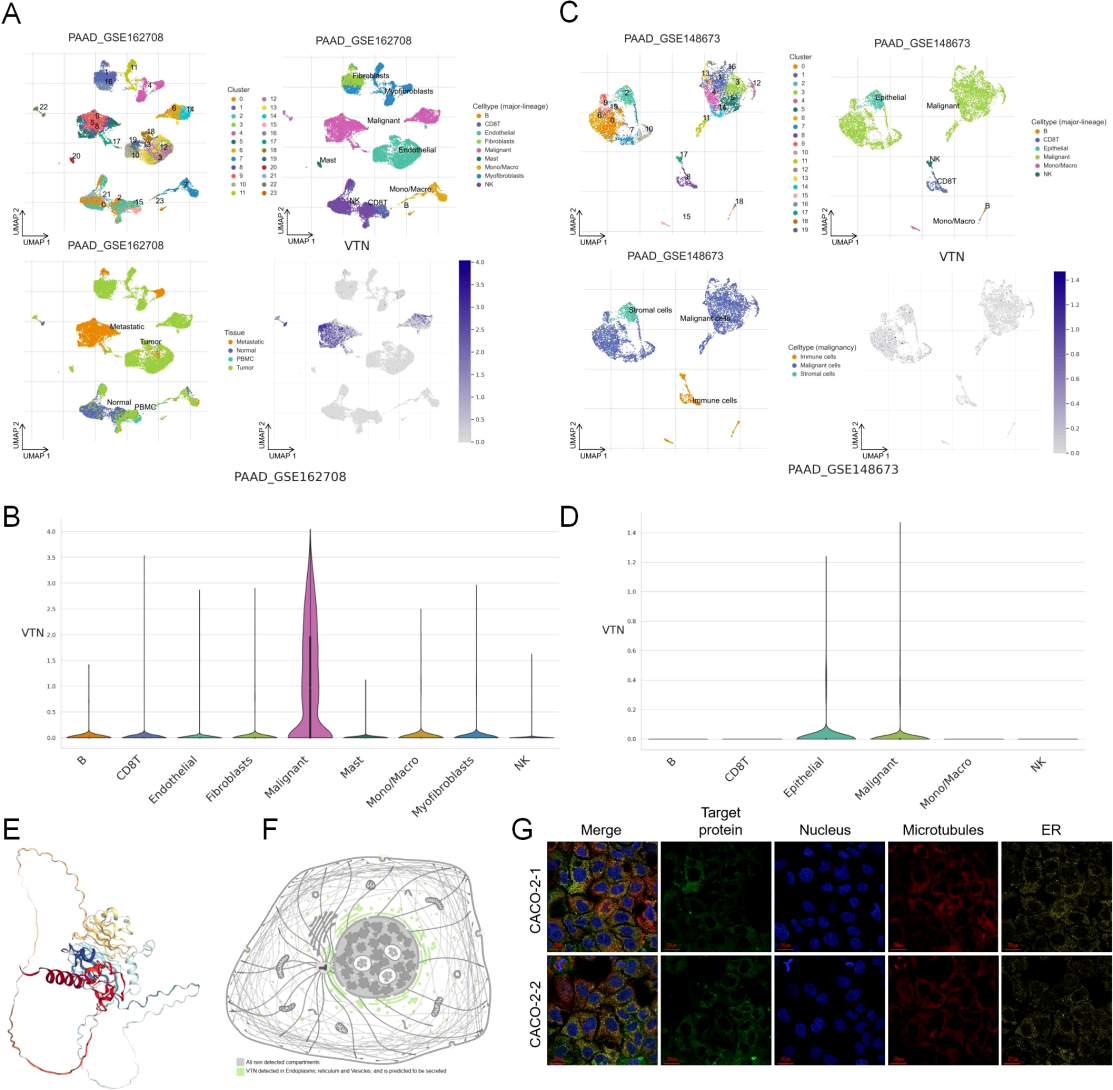
Single cell level analysis and subcellular localization of VTN in pancreatic cancer. **(A)** UMAP clustering of single-cell RNA sequencing data from the GSE1627089 dataset revealed nine distinct cell clusters in pancreatic cancer, including B cells, CD4+ T cells, CD8+ T cells, fibroblasts, malignant cells, and epithelial cells. **(B)** VTN expression was highly enriched in the malignant cell cluster. **(C)** UMAP clustering of the GSE148673 dataset identified six distinct cell clusters, including B cells, CD8+ T cells, NK cells, malignant cells, and epithelial cells. **(D)** In the GSE148673 dataset, VTN was predominantly expressed in both malignant and epithelial cell clusters. **(E)** The 3D structure of VTN. **(F)** The 2D structural diagram showing VTN detection in the endoplasmic reticulum and vesicles, with predictions indicating its secretion. **(G)** Fluorescence staining of VTN in two CACO-2 cell samples.

We analyzed the expression of VTN in pancreatic cancer using single-cell RNA sequencing data from two independent datasets, GSE1627089 and GSE148673. In the GSE1627089 dataset, UMAP clustering revealed a total of nine distinct cell clusters, including B cells, CD4+ T cells, CD8+ T cells, fibroblasts, malignant cells, and epithelial cells. Notably, VTN expression was highly enriched in the malignant cell cluster, with minimal expression observed in other cell types, including B cells, T cells, and fibroblasts (**Figures 1A, B**). In the GSE148673 dataset, which identified six distinct cell clusters, VTN was predominantly expressed in the malignant and epithelial cell clusters, while expression in other cell types, such as CD8+ T cells and NK cells, was minimal (**Figures 1C, D**). These findings suggest that VTN is specifically enriched in malignant and epithelial cells within the pancreatic tumor microenvironment. To investigate the subcellular localization of VTN, we utilized the Human Protein Atlas database. The 3D structure of VTN indicated the presence of specific functional domains that could be involved in tumor-related interactions (**Figure 1E**). The 2D structure showed that VTN is detected in the endoplasmic reticulum and vesicles, and is predicted to be secreted, aligning with its potential role in extracellular matrix remodeling and tumor progression (**Figure 1F**). Additionally, fluorescence staining of VTN in CACO-2 cells revealed VTN’s involvement in vesicular trafficking and secretion (**Figure 1G**).

### The expression of VTN was decreased in pancreatic cancer

**Figure 2:**
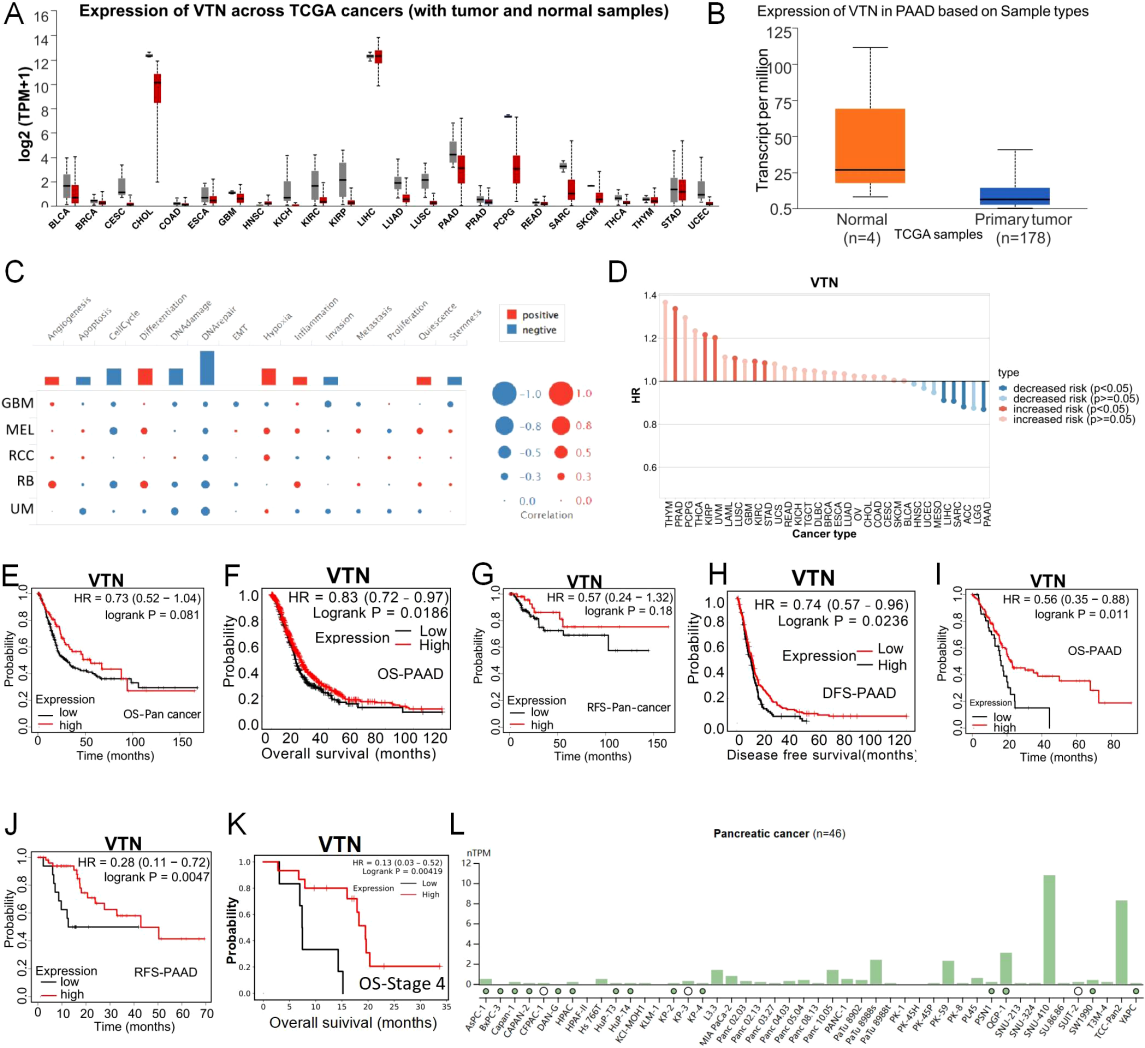
The expression of VTN was decreased in pancreatic cancer. **(A)** Expression of VTN mRNA in different cancers and corresponding normal tissues. **(B)** The expression of VTN mRNA in pancreatic cancer was significantly higher than that in normal tissue. **(C)** The functions of VTN in pancreatic cancer. **(D)** Relationship between VTN expression and risk of different cancers. **(C-F)** In pancarcinoma, patients with high expression of VTN have a better prognosis than those with low expression. **(G, H)** Base on gene chip, pancreatic cancer patients with high expression of VTN had longer OS and DFS than those with low expression. **(I, J)** Base on RNA-seq, pancreatic cancer patients with high expression of VTN had longer OS and RFS than those with low expression. **(K)** Pancreatic cancer patients at stage 4 with high expression of VTN had longer OS than those with low expression. **(L)** Expression of VTN in different pancreatic cancer cell lines.

Our initial investigations into the VTN gene’s expression in both normal and cancerous tissues were facilitated by databases such as GTEx and UALCAN. These studies revealed generally lower VTN expression levels in a variety of tumors when juxtaposed with their normal tissue equivalents (**Figure 2A**). Further analysis confined to pancreatic cancer substantiated a significant repression in VTN expression in tumor tissues versus normal ones (**Figure 2B**), with a noted downtrend accompanying the progression of the disease, especially marked in stage 4 pancreatic cancer (**Supplementary Figure S1A**). Cross-referencing the VTN expression across different tumor grades yielded a consistent observation (**Supplementary Figure S1B**). Lifestyle influences, particularly alcohol consumption, were identified as determinants of VTN expression; those with a history of alcohol use showing lower levels of VTN (**Supplementary Figure S1C**). The expression of VTN was further influenced by a history of chronic pancreatitis, with past sufferers showing reduced levels (**Supplementary Figure S1D**). Equally, a comparison between TP53-Mutant and TP53-NonMutant patients showed reduced VTN expression for the former group (**Supplementary Figure S1E**). In order to investigate the functions of VTN in pancreatic cancer, single-cell analysis was performed using CancerSEA. The results indicated that VTN may negatively regulate tumor apoptosis, cell cycle, DNA damage repair, invasion, and stemness, and may positively regulate tumor Angiogenesis, differentiation, hypoxia, apoptosis, apoptosis, and inflammation (**Figure 2C**).

### VTN can be used as a prognostic indicator in pancreatic cancer patients

Furthermore, we utilized the TISCH database to further analyze the relationship between VTN and the risk of different cancer types, and the results indicated a negative correlation between VTN expression and the risk of pancreatic cancer (**Figure 2D**). Leveraging the Kaplan-Meier Plotter, we dove into the link between VTN expression and the prognosis of patients. Our data revealed that individuals exhibiting elevated VTN expression in pancarcinoma have more favorable prognoses compared to their low-expression counterparts (**Figures 2E, F**). This pattern holds true, as corroborated by gene chip and RNA-seq analyses among pancreatic cancer patients (**Figures 2G-J**). Data from TCGA analyses underline that reduced VTN expression is aligned with more advanced tumor stages: a particularly stark prognosis difference was observed in stage 4 pancreatic cancer patients, where low VTN expression was connected to significantly worse outcomes than seen with high expression levels (**Figure 2K**). These findings bolster the idea that VTN expression may have predictive value regarding the course of pancreatic cancer. We utilized the Human Protein Atlas database to detect the expression of VTN in different pancreatic cancer cell lines, and selected PANC1 with low expression of VTN and PATU8988 with medium expression of VTN for subsequent experiments (**Figure 2L**).

### Over-expression of VTN suppressed the proliferation, invasion and migration ability of human pancreatic cancer cell lines

**Figure 3:**
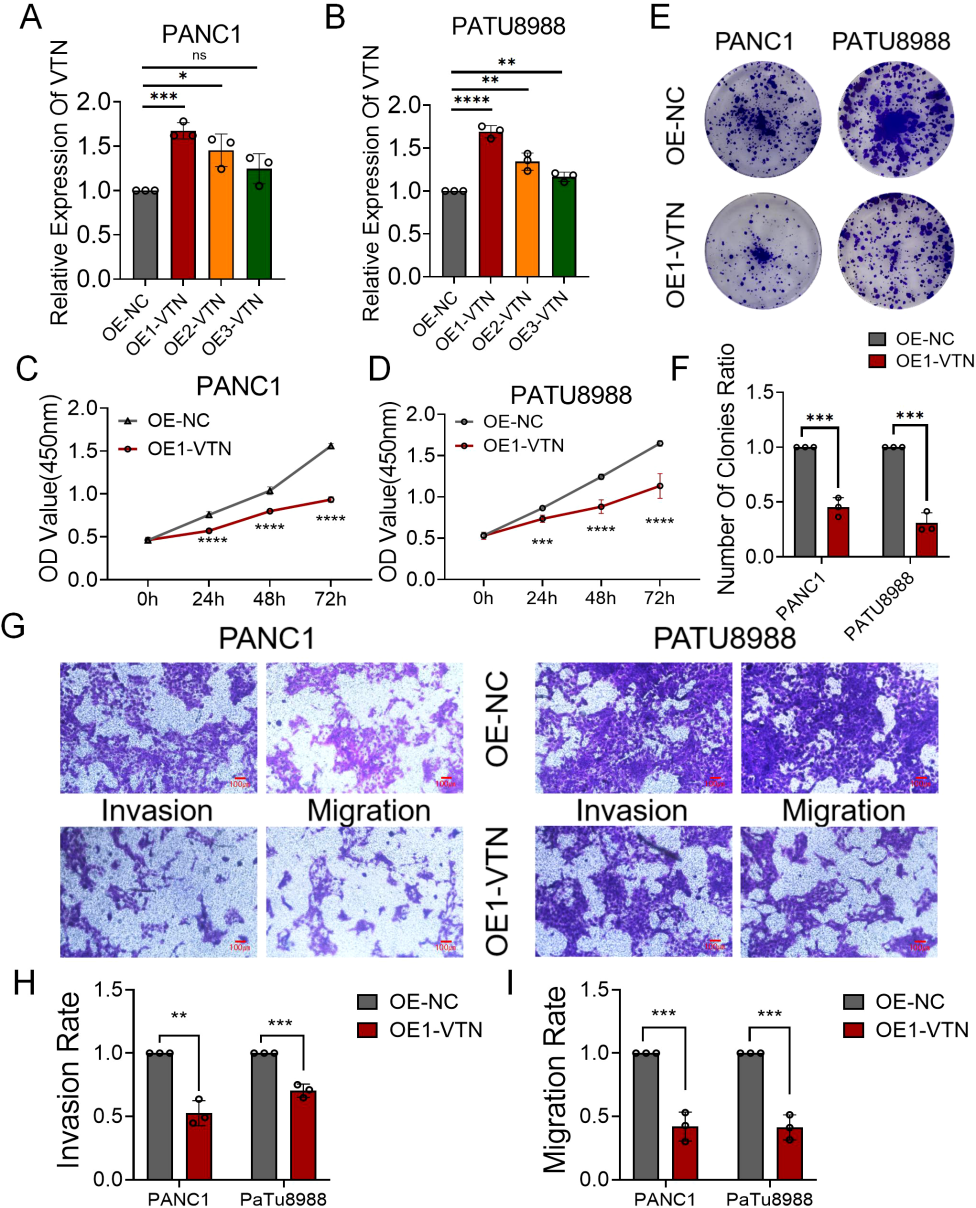
VTN knockdown promoted the proliferation, invasion and migration ability of human pancreatic cancer cell lines. **(A, B)** Three siRNAs (si1, si2, and si3) were designed to silence VTN in pancreatic cancer cells (PANC1 and PATU8988), and validated by qRT-PCR. **(C, D)** The growth curves of PANC1 **(C)** and PATU8988 **(D)** cells were plotted after transfection with si1-VTN/si-NC based on CCK-8 assay. **(E, F)** Colony formation assays demonstrated that knockdown of VTN promoted the proliferation of PANC1 and PATU8988 cells. **(G-I)** Transwells experiment demonstrated that knockdown of VTN expression could effectively promote the migration and invasion ability of pancreatic cancer cells. ns: p>0.05, *: p<0.05, **: p<0.01, ***: p<0.001, ****: p<0.0001.

In a concerted effort to elevate VTN gene expression, we engineered and deployed three distinct VTN-targeting lentiviruses into mouse renal cancer cell lines. Our qRT-PCR results spotlighted the viral construct OE1-VTN as the most efficient at amplifying VTN levels (**Figures 3A, B**). Observations from CCK8 proliferation assays indicated a pronounced reduction in the growth rate of pancreatic cancer cells upon VTN overexpression (**Figures 3C, D**). This inhibitory effect on cell proliferation was further substantiated by colony formation assays, with data hinting at VTN’s role in the moderation of cellular expansion (**Figures 3E, F**). Additionally, transwell assays measuring invasion and migration capabilities presented a noteworthy decrease in both activities across PANC1 and PATU8988 cells in the context of heightened VTN expression (**Figures 3G–I**). Together, these experiments underline the potential of VTN expression as a mitigating factor in the progression of pancreatic cancer.

### VTN knockdown promoted the proliferation, invasion and migration ability of human pancreatic cancer cell lines

**Figure 4:**
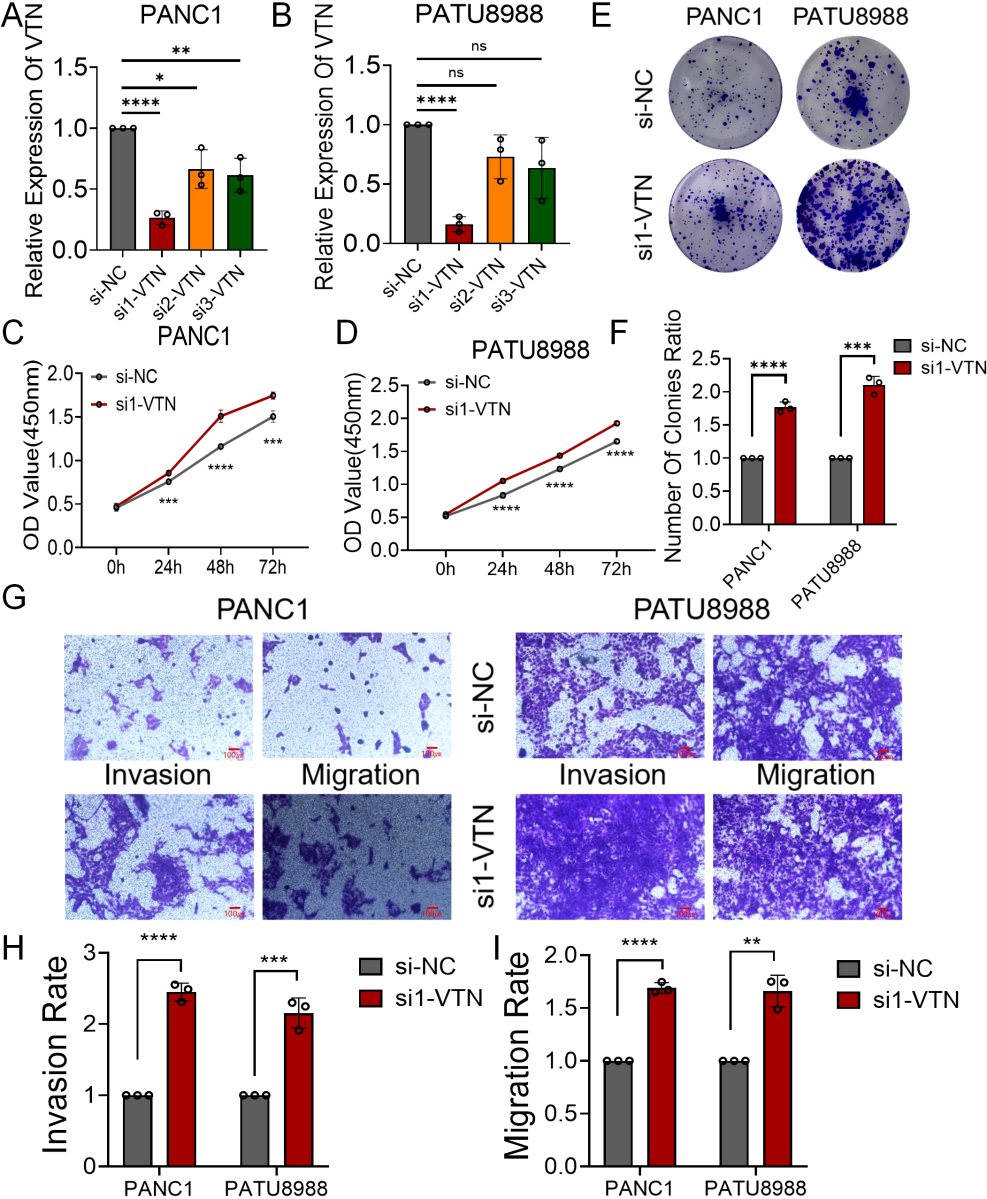
Overexpression of VTN inhibited proliferation, invasion and migration of human pancreatic cancer cell lines. **(A, B)** qRT-PCR verified the efficiency of overexpression of VTN in PANC1 and PATU8988 cell lines. **(C, D)** The growth curves of PANC1 **(C)** and PATU8988 **(D)** cells were plotted after overexpression of VTN based on CCK-8 assay. **(E, F)** Colony formation assays demonstrated that overexpression of VTN inhibited the proliferation of PANC1 and PATU8988 cells. **(G-I)** Transwells experiment demonstrated that overexpression of VTN could effectively inhibit the migration and invasion ability of pancreatic cancer cells.. ns: p>0.05, *: p<0.05, **: p<0.01, ***: p<0.001, ****: p<0.0001.

We synthesized three si-RNAs specifically targeting the VTN gene to modulate its expression within PANC1 and PATU8988 cell lines. Our qRT-PCR analysis identified si1-VTN as the most potent in diminishing VTN expression, justifying its selection for subsequent studies (**Figures 4A, B**). Further investigation through CCK8 assays demonstrated a significant upsurge in cell proliferation of both PANC1 and PATU8988 cell lines following VTN knockdown (**Figures 4C, D**). These findings were echoed in the colony formation assays, which reflected similar proliferative increases in these cell lines (**Figures 4E, F**). Additionally, transwell assays provided insight into the migration and invasion capabilities, which were markedly elevated after VTN reduction (**Figures 4G-I**). This evidence supports the hypothesis of VTN’s potential anti-cancer properties in the context of pancreatic cancer.

### VTN can be used as a predictor of immunotherapy effect in pancreatic cancer patients

**Figure 5:**
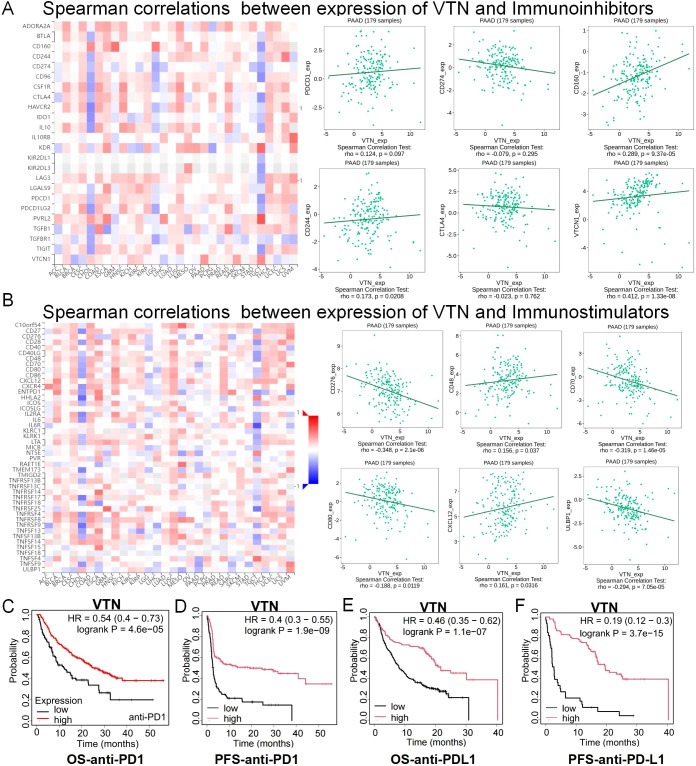
The expression of VTN was correlated with immunomodulator. **(A)** Correlation between VTN and immunoinhibitors in different cancers. **(B)** Correlation between VTN and immunostimulator in different cancers. **(C, F)** In patients treated with anti-PD1 **(C, D)** and anti-PD-L1 **(E, F)**, high expression of VTN has a better prognosis.

These results arouse our interest in the role of VTN in tumor immunity. We used TISIDB, an integrated repository portal for tumor-immune system interactions, to analyze the relationship between VTN and various immune regulatory factors in different tumors. In pancreatic cancer, VTN is associated with multiple immunoinhibitor factors (**Figure 5A**), immunostimulators (**Figure 5B**), and MHCs (**Supplementary Figure S2A**). On the one hand, VTN was positively correlated with immunosuppressive factors such as CD244, CD160 and VTCN1. On the other hand, it was also positively correlated with CD48, CXCL12 and other immunostimulatory factors. Notably, although not statistically significant, there was a trend of positive correlation between VTN and PDCD1. However, there was a negative correlation between VTN and some classical immunosuppressive modulators, such as PD-L1 and CTLA4, although there was also no statistical significance. It is suggested that VTN may play a very complex role in immune regulation. Although our *in vivo* experiments in **Figures 3**, **4** show that VTN itself plays an important role in cancer suppression in pancreatic cancer, this part of the research results suggest that VTN may also regulate the progression of pancreatic cancer by regulating the tumor immune microenvironment. This is worthy of further exploration in our future research. At the same time, the correlation between VTN and immunomodulatory factors also suggests that we should further explore the correlation between VTN and immunotherapy efficacy. Our analysis indicates that patients with robust VTN expression could be more receptive to immunotherapy. Notably, patients with heightened VTN levels demonstrated better prognoses post-treatment with anti-PD1 (**Figures 5C, D**) and anti-PD-L1 therapies (**Figures 5E, F**). Although the above results suggest that there may be a certain correlation between VTN expression and the efficacy of immunotherapy, it is noteworthy that these results cannot exclude the survival benefit brought by high VTN expression itself. Therefore, these results only indicate that high VTN expression may provide survival benefits to patients receiving immunotherapy. This also prompts us to further explore the relationship between VTN and immunotherapy.

### Overexpression of VTN inhibits pancreatic cancer growth and enhances anti-PD1 efficacy

**Figure 6:**
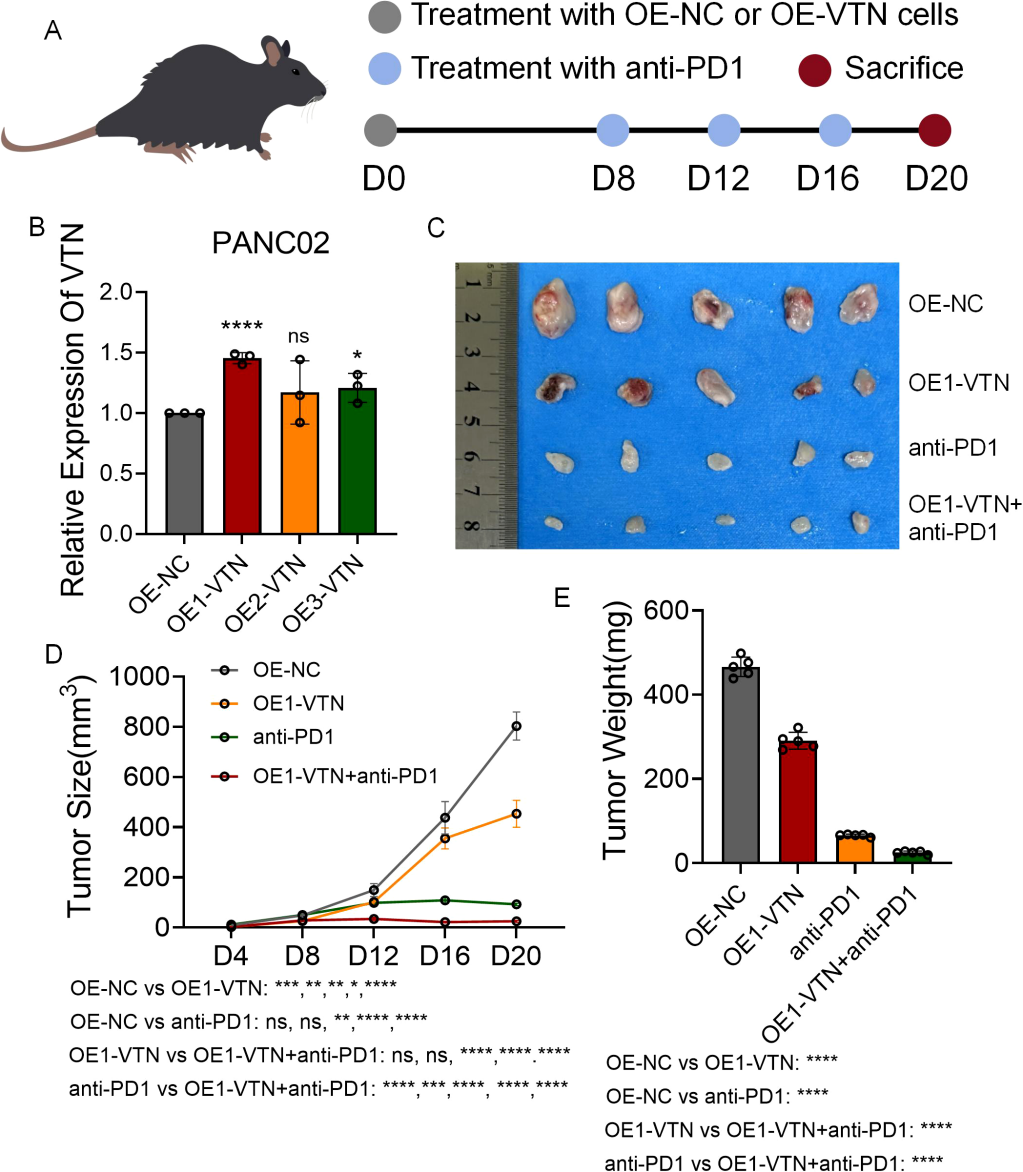
Overexpression of VTN inhibits pancreatic cancer growth and enhances anti-PD1 efficacy. **(A)** Schematic diagram of establishment of mouse subcutaneous tumor model in each group. **(B)** Three lentiviruses were designed to overexpress VTN expression in PANC02 cell lines, and their overexpression efficiency was verified by qRT-PCR. **(C)** Images of subcutaneous tumors in each group. **(D, E)** Analysis of subcutaneous tumors in the respective groups. ns: p>0.05, *: p<0.05, **: p<0.01, ***: p<0.001, ****: p<0.0001.

To probe the impact of VTN on immunotherapeutic outcomes, we synthesized three distinct lentiviral vectors to enrich VTN expression in mouse renal cell carcinoma cultures (**Figure 6A**). Through qRT-PCR analysis, it was determined that OE1-VTN excelled in maximizing VTN expression (**Figure 6B**). Developing a mouse subcutaneous tumor model with both normal and VTN-overexpressing PANC02 cells, the data illustrated a notable suppression in tumor growth rate following VTN induction. Furthermore, mice with VTN-overexpressed tumors treated with anti-PD1 agents showed a considerable slowdown in tumor growth compared to those receiving anti-PD1 treatment or VTN overexpression independently (**Figures 6C-E**). These findings reaffirmed VTN’s potential as an anti-tumor agent with enhanced sensitivity to anti-PD1 immunotherapy.

## Discussion

VTN is ubiquitously present in human plasma and the extracellular matrix (42), our findings suggest that its decline in pancreatic cancer tissues is predominantly driven by intrinsic downregulation within malignant cells rather than systemic depletion in peripheral blood. Single-cell RNA sequencing revealed that VTN expression is highly enriched in malignant and epithelial cell clusters, with minimal contributions from stromal or immune cells. This tumor cell-specific localization aligns with our observation that VTN levels are significantly reduced in pancreatic cancer tissues compared to adjacent normal tissues and correlate inversely with disease progression. Functional experiments further support this hypothesis: cancer cell-intrinsic VTN knockdown enhanced proliferation, invasion, and migration, while overexpression suppressed tumor growth and synergized with anti-PD1 therapy. These results imply that VTN loss in pancreatic cancer is a localized, tumor-driven phenomenon rather than a systemic deficit. However, we acknowledge that our study did not directly measure circulating VTN levels or dissect potential contributions from stromal cells. Future studies should investigate whether plasma VTN levels parallel tissue expression and explore how tumor-derived VTN interacts with systemic pools to shape the immunosuppressive microenvironment. Such insights would clarify the dual role of VTN as both a tissue-specific tumor suppressor and a plasma-borne modulator of immunity.

Previous studies predominantly demonstrate that VTN is an oncogenic factor that advances tumor progression in a variety of malignancies, including breast and ovarian cancers, through multiple mechanisms. VTN enhances the adhesion ability of cancer cells to vasculature and tissues, facilitating metastasis and invasion within the body. Moreover, VTN interacts with integrins on the surface of tumor cells to ward off apoptotic signals, fostering cell survival and proliferation, thereby expediting tumor progression. VTN also plays a pivotal role in the migration of endothelial cells and the formation of new blood vessels, supplying tumors with essential nutrients and oxygen, all of which underscore its contributory role in oncogenesis (43–45). However, our research indicates a paradoxical role for VTN in pancreatic cancer. On one hand, overexpression of VTN in pancreatic cancer cell lines significantly hinders cellular proliferation, invasion, and migration. On the other hand, *in vivo* studies suggest that pancreatic cancer cell lines with upregulated VTN exhibit increased sensitivity to anti-PD1 therapy. Additionally, pancreatic cancer data from Kaplan-Meier Plotter corroborate our findings, showing improved prognosis for patients with high VTN expression and better therapeutic outcomes when treated with anti-PD1 or anti-PD-L1. These insights suggest that VTN’s role in tumorigenesis may be multifaceted. This may be due to the unique pancreatic tumor microenvironment (TME) and signaling rewiring (46, 47). Pancreatic cancer is characterized by a dense desmoplastic stroma rich in extracellular matrix (ECM) components like collagen and fibronectin, which promote tumor progression and immune evasion (47). VTN downregulation in pancreatic cancer tissues may disrupt ECM remodeling processes that sustain stromal stiffness. For instance, VTN antagonizes fibronectin-integrin interactions (47), and its restoration (via overexpression) could mitigate stromal fibrosis, impairing invasion while enhancing T-cell infiltration (48, 49). The signals through αvβ3 and αvβ5 integrins were required for VTN-promoted hematopoietic differentiation (50). However, pancreatic cancer predominantly expresses αvβ6 integrins, which drive TGF-β-mediated EMT (51–53). VTN in pancreatic cancer may compete with TGF-β ligands for integrin binding, suppressing EMT and stromal crosstalk, as evidenced by reduced invasion/migration upon VTN overexpression. While VTN suppresses complement-mediated inflammation (54), it positively correlates with immunostimulatory factors (CXCL12). This dual role aligns with its ability to enhance anti-PD1 efficacy *in vivo*4, suggesting VTN reshapes the immunosuppressive TME by balancing immune activation and suppression. Frequent deletion of the VTN locus in pancreatic cancer and its association with advanced disease imply that VTN loss confers a survival advantage by promoting immune evasion. However, this adaptation may sensitize tumors to immunotherapy when VTN is restored, as shown in combination therapy models. These findings may suggest how pancreatic cancer biology and TME-specific interactions determine the environment-dependent effects of VTN.

Our study provides the evidence that VTN acts as a dual regulator of pancreatic cancer progression and anti-PD1 immunotherapy sensitivity. While prior research has focused on VTN’s roles in cell adhesion and metastasis in other cancers, our single-cell and bulk RNA-seq analyses uniquely identified its tumor-suppressive expression pattern in pancreatic malignancies. Critically, VTN expression correlated with key immunomodulators: positive associations with CXCL12 (a chemokine promoting T-cell recruitment) and negative trends with PD-L1/CTLA4 (immune checkpoint mediators) suggest that VTN may remodel the tumor immune microenvironment. This hypothesis is functionally supported by our *in vivo* findings, where VTN overexpression not only suppressed tumor growth but also synergized with anti-PD1 to amplify therapeutic efficacy. The enhanced response likely stems from VTN’s dual modulation of tumor-intrinsic and immune-related pathways. For instance, VTN loss in pancreatic cancer may promote stromal fibrosis via dysregulated integrin signaling, fostering an immunosuppressive TME resistant to T-cell infiltration. Restoring VTN expression could disrupt this fibrotic niche, as evidenced by reduced invasion/migration *in vitro* and delayed tumor progression *in vivo*, thereby sensitizing tumors to checkpoint blockade. Although the precise mechanisms linking VTN to PD1/PD-L1 require further investigation, our data position VTN as both a prognostic biomarker for immunotherapy response and a therapeutic target to overcome resistance in pancreatic cancer. These findings contrast with VTN’s pro-tumor roles in other cancers, highlighting tissue-specific ECM-immune crosstalk as a determinant of therapeutic outcomes.

Immuno-oncological modalities such as PD1 and PD-L1 inhibitors have been corroborated by an extensive body of research as being effective in the treatment of pancreatic cancer. A seminal study conducted in 2022 elucidated the efficaciousness and safety of a therapeutic synergy involving sotigalimab and/or nivolumab alongside chemotherapy in the frontline management of metastatic pancreatic cancer. However, consonant with the narrative in other malignancies, the real-world application yields benefit to only a select cohort of patients. Contemporary research endeavors are increasingly dedicated to amplifying the sensitivity of cancer cells to immunotherapeutic interventions. Empirical data has evidenced that the strategic inhibition of targets such as Pin1, MEK, STAT3, ARG1 can significantly elevate the response rates of pancreatic neoplasms to anti-PD1 therapy (55, 56), enable more pancreatic cancer patients to benefit from immunotherapy. Our research delineates that VTN can serve as a prognostic indicator for the efficacy of anti-PD1/PD-L1 therapies. Patients exhibiting an elevated expression of VTN display heightened sensitivity to these immunotherapeutic approaches and consequentially have a more favorable prognosis. Moreover, by leveraging a lentivirus to boost VTN expression within the PANC02 pancreatic cancer cell line in mice and subsequently crafting a subcutaneous tumor model, we observed an amplified therapeutic response when coupled with anti-PD1 administration. Our findings reveal that VTN’s antitumor activity in pancreatic cancer is mediated, at least in part, through its multifaceted interactions with immune regulatory factors. The observed positive correlation between VTN and immunostimulants such as CXCL12, a chemokine critical for T cell recruitment, potentially suggests that overexpression of VTN may trigger an enhanced anti-tumor immune response in tumors. Conversely, VTN was negatively correlated with PD-L1 and CTLA4 (a key mediator of t cell failure) and positively correlated with PD1, indicating the complex regulatory function of VTN on immunosuppressive signaling pathways and the potential ability of VTN to sensialize tumors to pd1 therapy. This dual regulatory capacity is consistent with prior studies demonstrating VTN’s role in modulating complement-mediated inflammation and integrin-dependent immune cell adhesion (54, 57). Importantly, our *in vivo* data suggest that VTN overexpression synergistically enhances anti-tumor response with anti-PD1, providing functional validation of its immune-potentiating effects. While the precise molecular mechanisms require further exploration, our results tentatively establish VTN as a critical nexus between tumor progression and immune evasion in pancreatic cancer.

In closing, this study postulates that VTN constitutes a latent prognostic marker and therapeutic target for pancreatic carcinoma, as well as a predictive and potentiating target for the efficacy of anti-PD1 interventions. Nevertheless, the research is not without its limitations; our explorations did not extend to an in-depth validation using VTN gene-deficient murine models or patient-derived xenografts (PDX), nor did we probe into the intricate molecular interplay between VTN and PD1. These aspects are earmarked for comprehensive scrutiny in our forthcoming research initiatives.

## Data Availability

The datasets presented in this study can be found in online repositories. The names of the repository/repositories and accession number(s) can be found below: https://www.ncbi.nlm.nih.gov/geo/, GSE1627089; https://www.ncbi.nlm.nih.gov/geo/, GSE148673.

## Supplementary material

Supplementary Figure 1 Expression profile of VTN in pancreatic cancer. **(A)** Relationship between VTN expression and tumor stage in pancreatic cancer. **(B)** Relationship between VTN expression and tumor grade in pancreatic cancer. **(C)** Relationship between VTN expression and alcohol consumption in pancreatic cancer. **(D)** Relationship between VTN expression and history of chronic pancreatitis in pancreatic cancer. **(E)** Relationship between VTN expression and TP53 mutant in pancreatic cancer.

Supplementary Figure 2 The expression of VTN was correlated with MHCs.
