# Supplementary figures and images for: Reversing VTN deficiency inhibits the progression of pancreatic cancer and enhances sensitivity to anti-PD1 immunotherapy
Source: Front Immunol. 2025 May 13;16:1578870. doi: 10.3389/fimmu.2025.1578870 (PMC12106453; doi:10.3389/fimmu.2025.1578870)

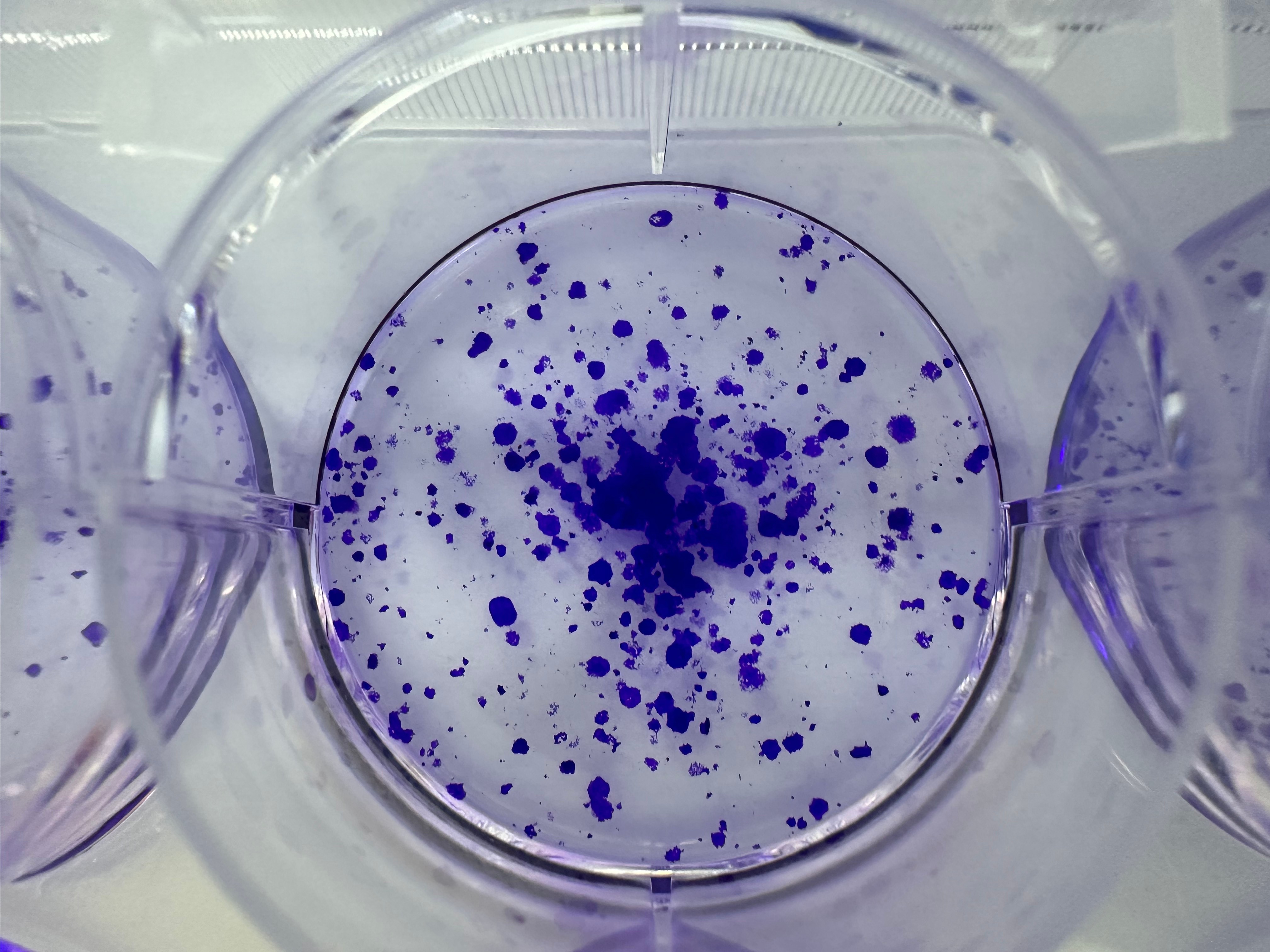

Supplement: Supplementary file 1 [file DataSheet1.zip › Raw image data_Plate cloning/PANC1/OE-NC/V41.jpg]

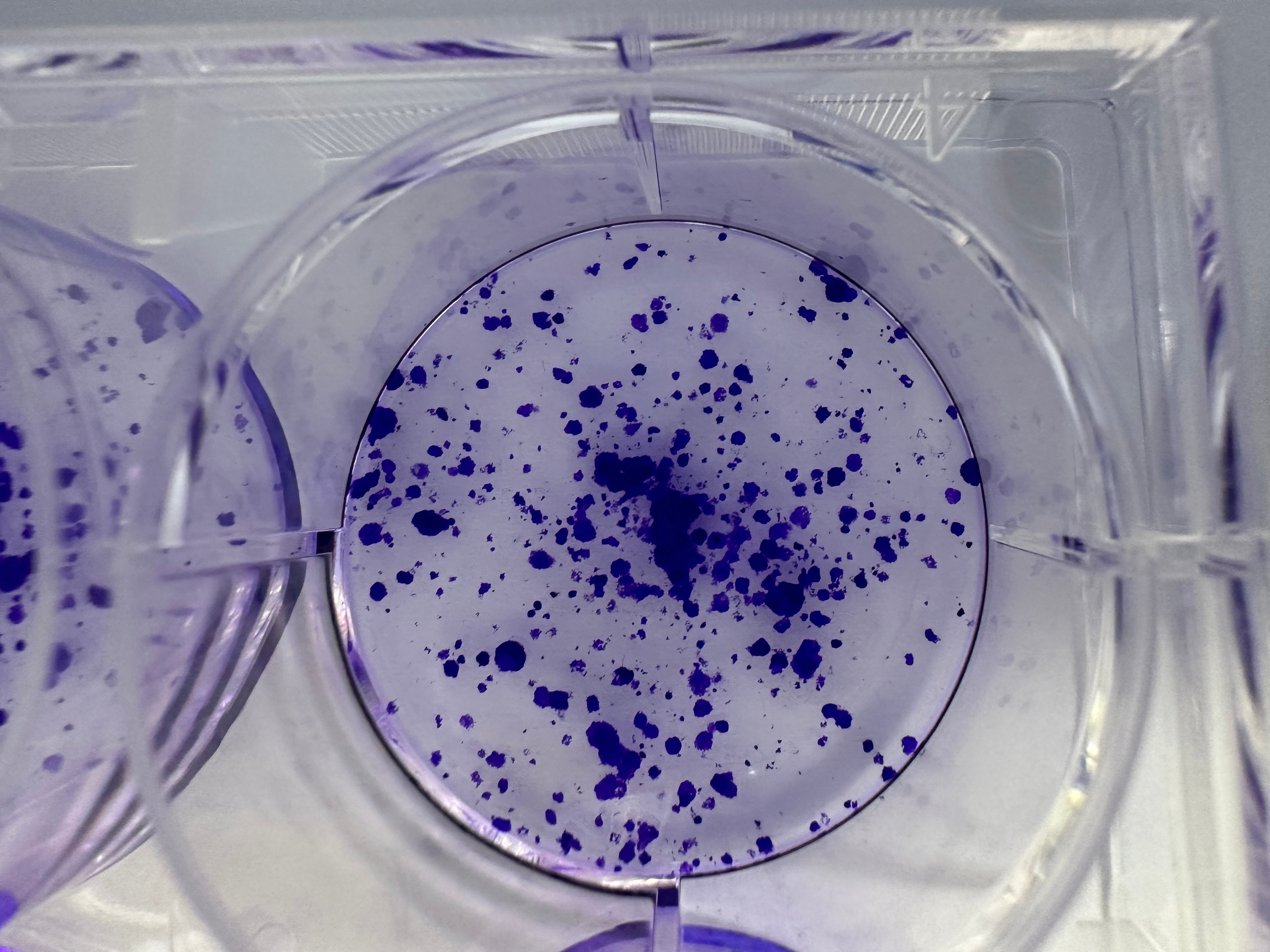

Supplement: Supplementary file 1 [file DataSheet1.zip › Raw image data_Plate cloning/PANC1/OE-NC/V42-3.jpg]

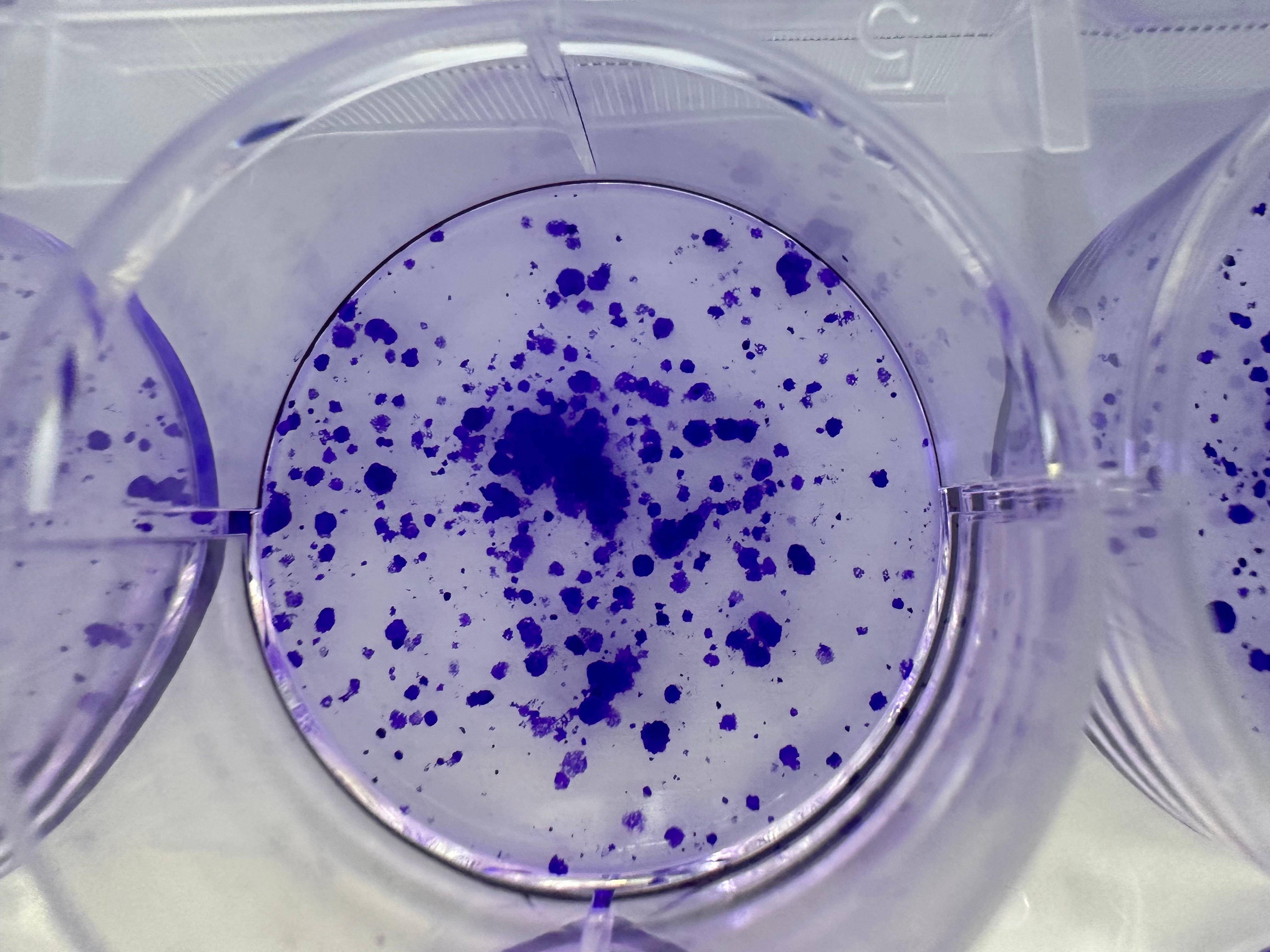

Supplement: Supplementary file 1 [file DataSheet1.zip › Raw image data_Plate cloning/PANC1/OE-NC/V43.jpg]

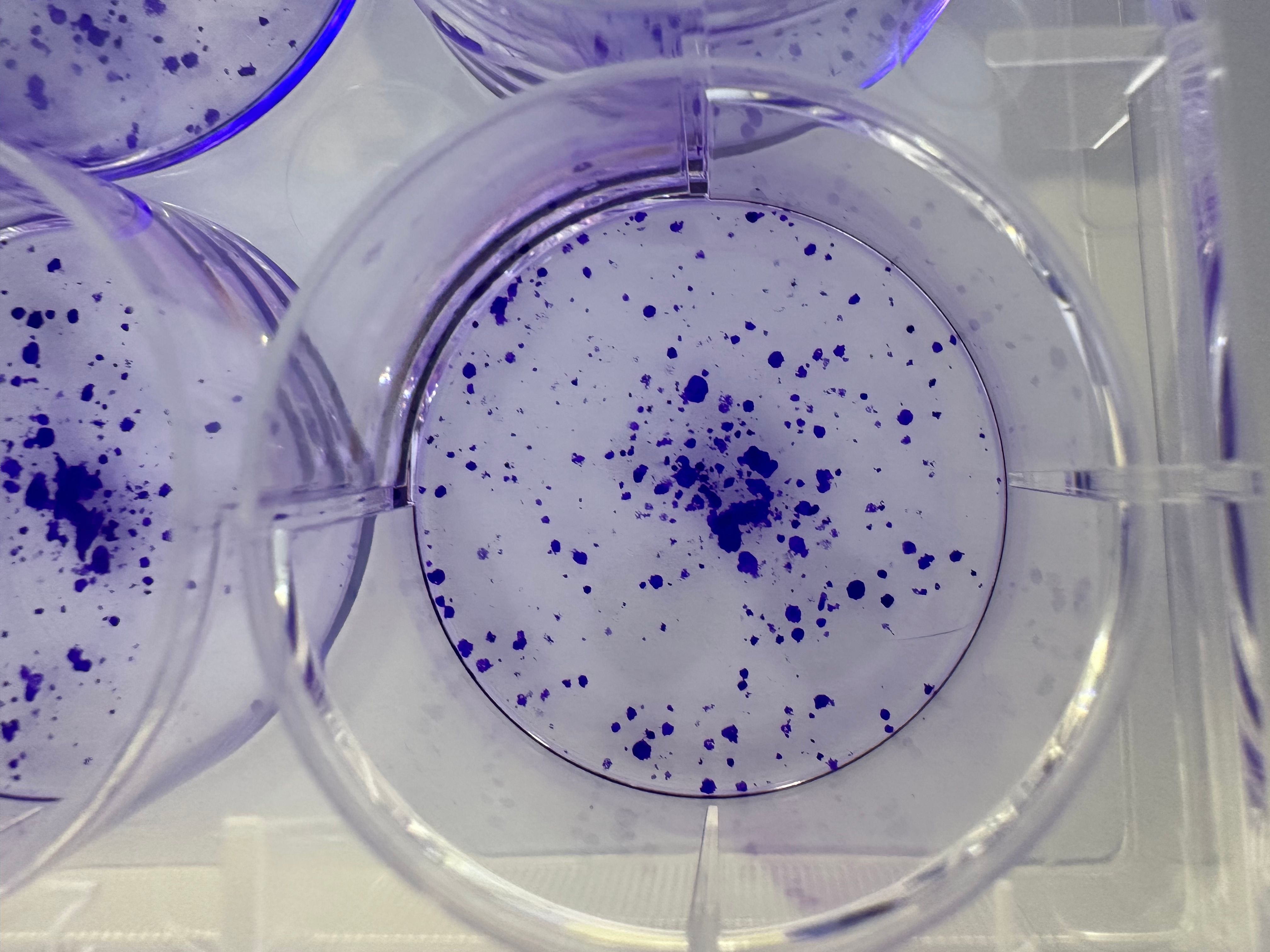

Supplement: Supplementary file 1 [file DataSheet1.zip › Raw image data_Plate cloning/PANC1/OE-VTN/V31.jpg]

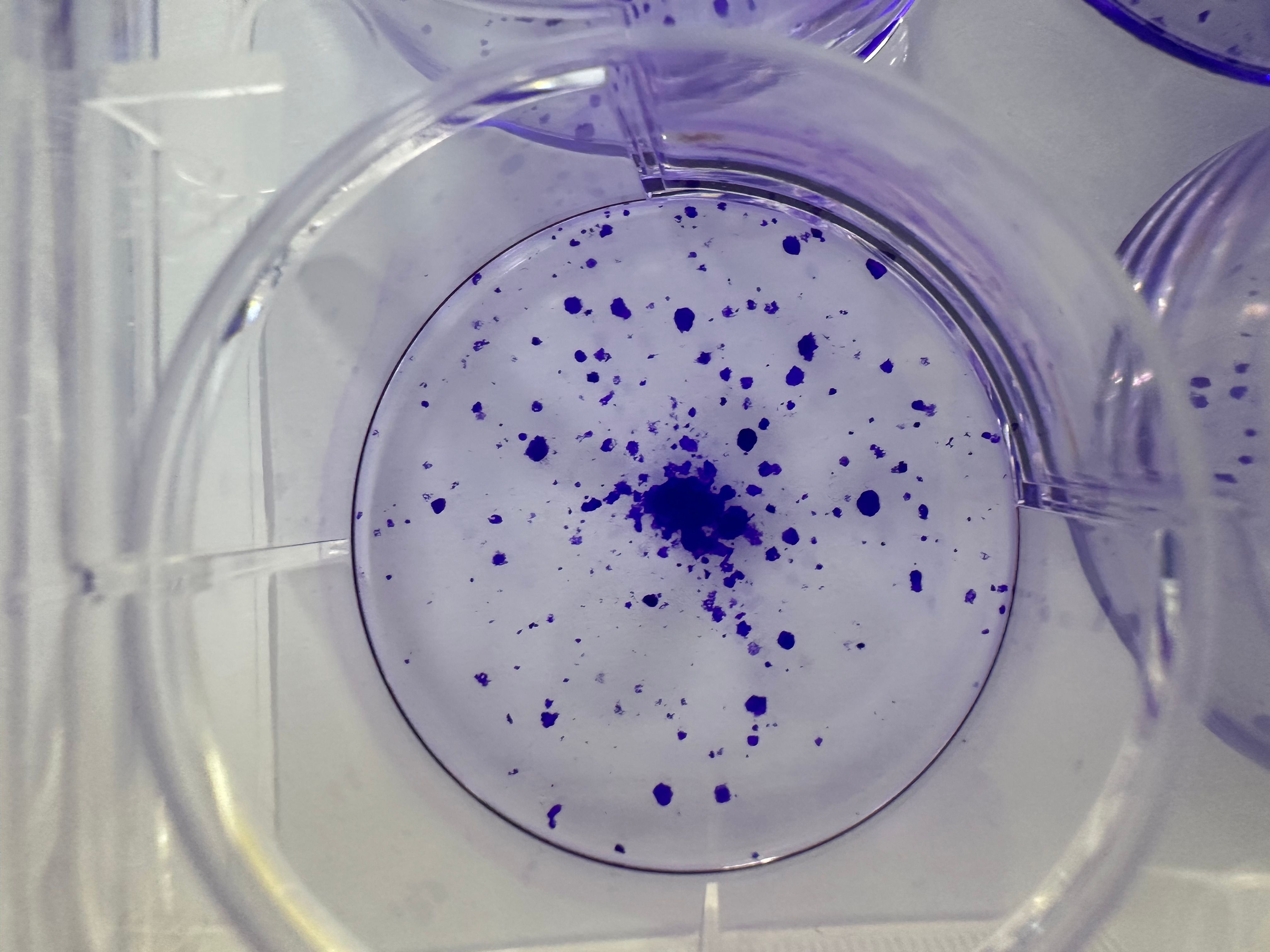

Supplement: Supplementary file 1 [file DataSheet1.zip › Raw image data_Plate cloning/PANC1/OE-VTN/V32-4.jpg]

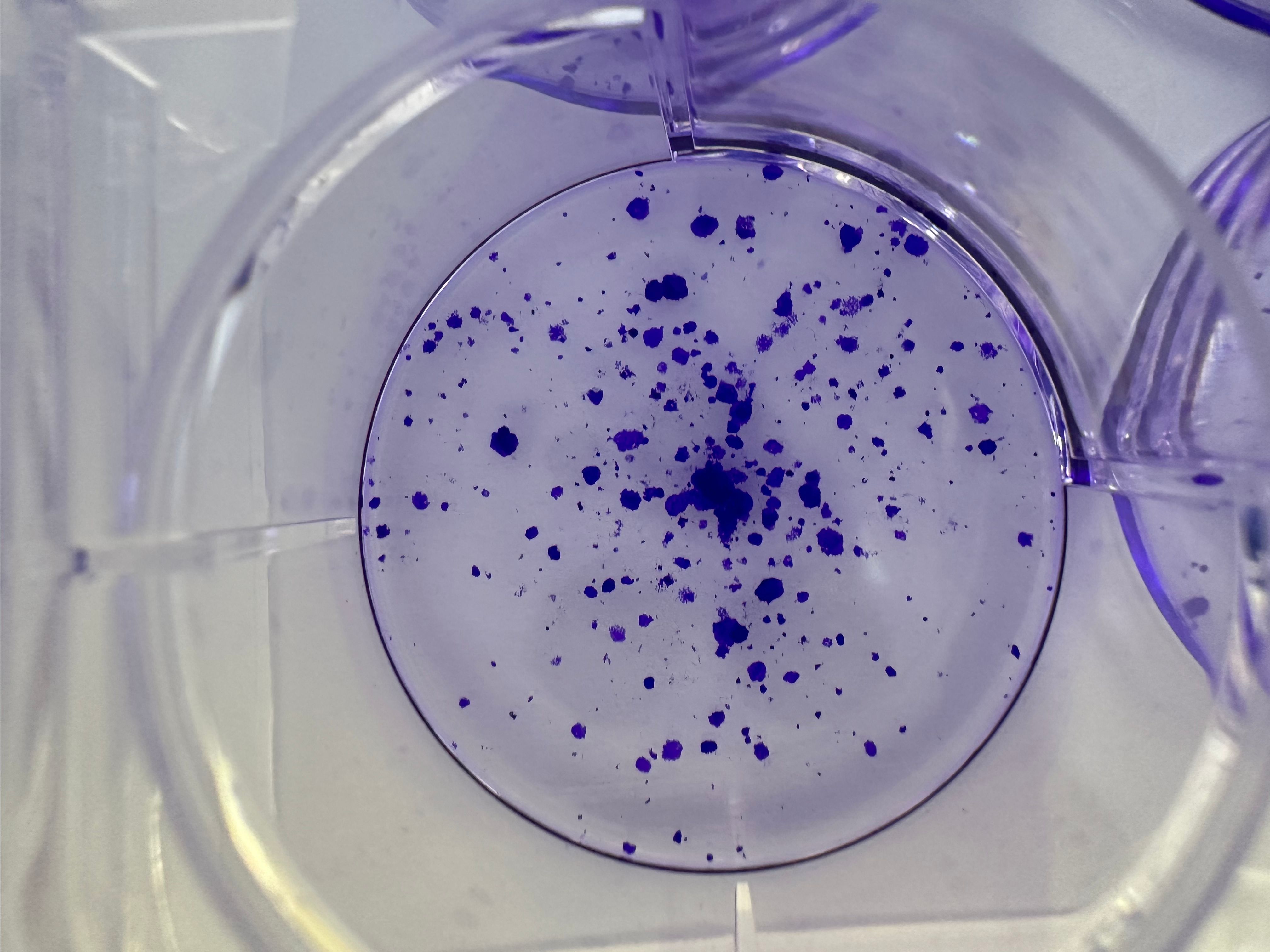

Supplement: Supplementary file 1 [file DataSheet1.zip › Raw image data_Plate cloning/PANC1/OE-VTN/V33.jpg]

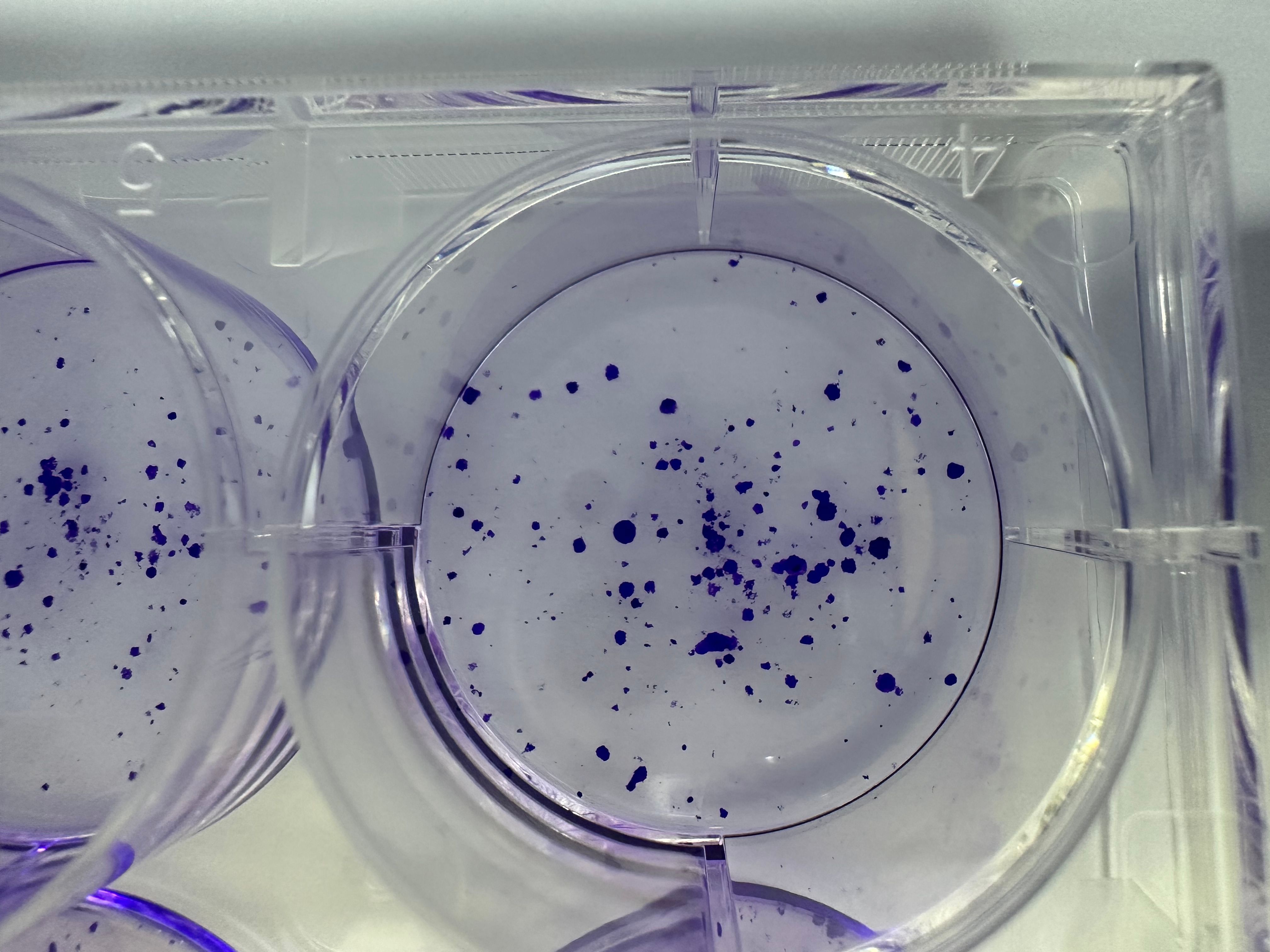

Supplement: Supplementary file 1 [file DataSheet1.zip › Raw image data_Plate cloning/PANC1/si-NC/V21.jpg]

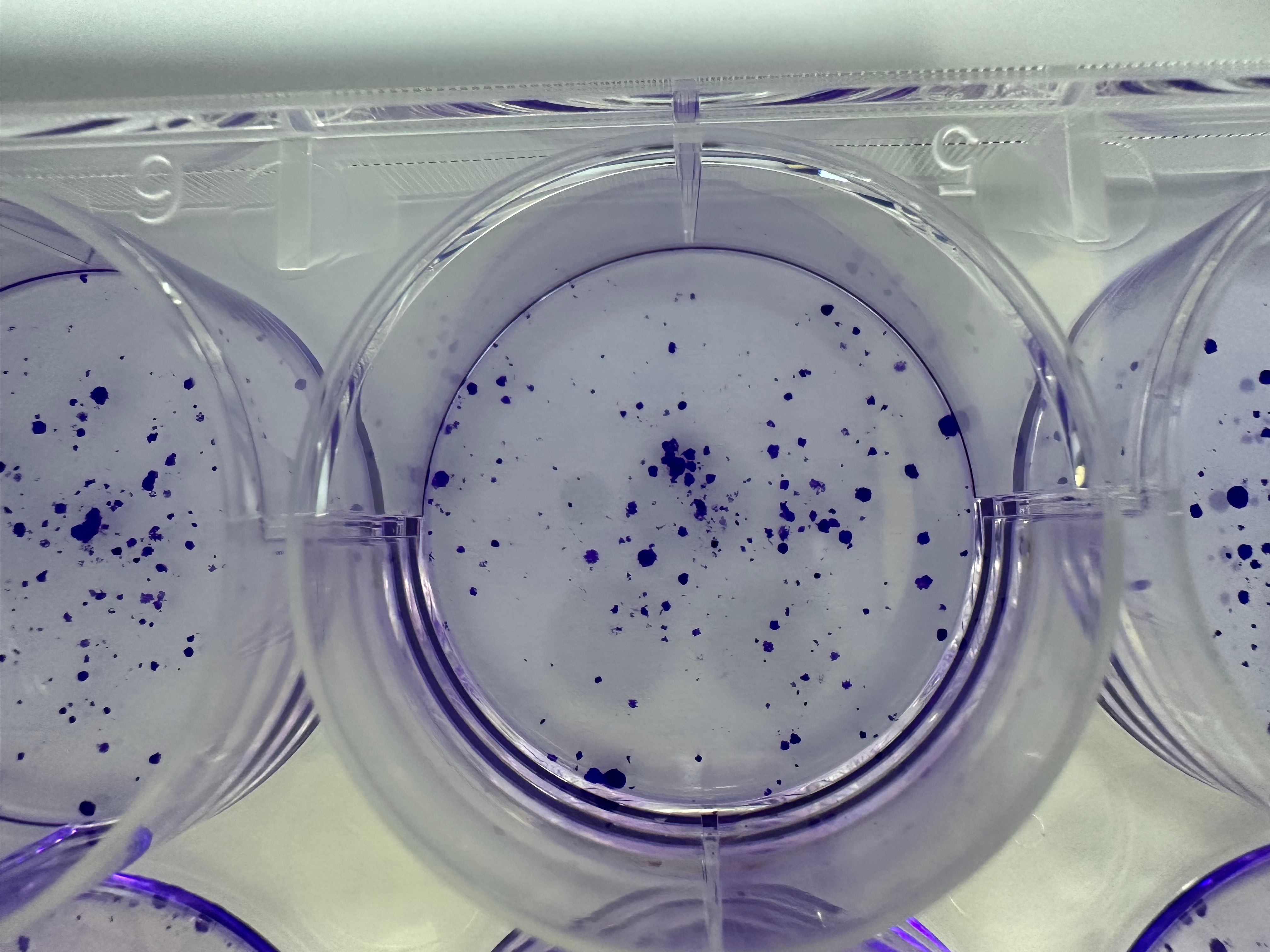

Supplement: Supplementary file 1 [file DataSheet1.zip › Raw image data_Plate cloning/PANC1/si-NC/V22.jpg]

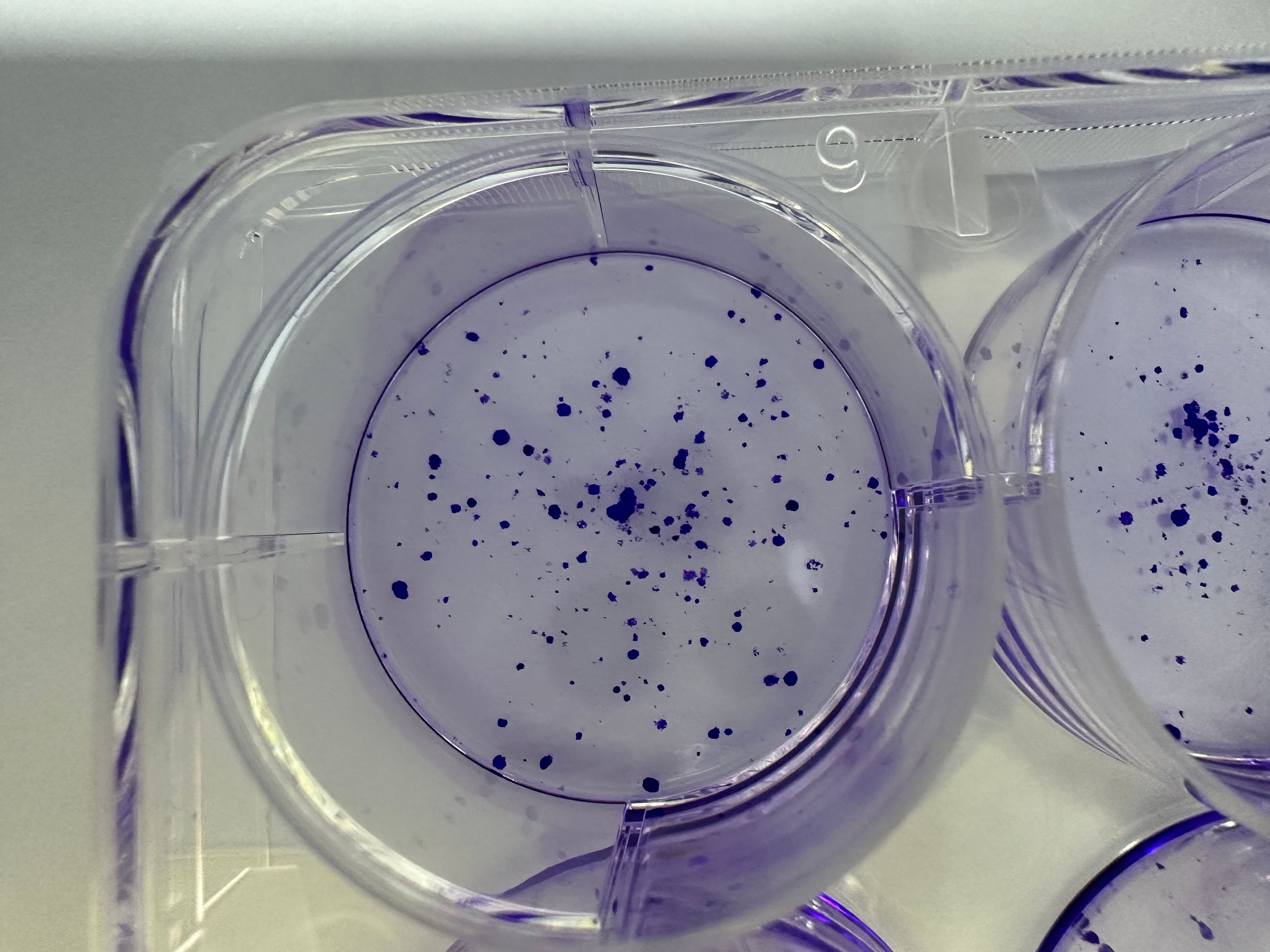

Supplement: Supplementary file 1 [file DataSheet1.zip › Raw image data_Plate cloning/PANC1/si-NC/V23-1.jpg]

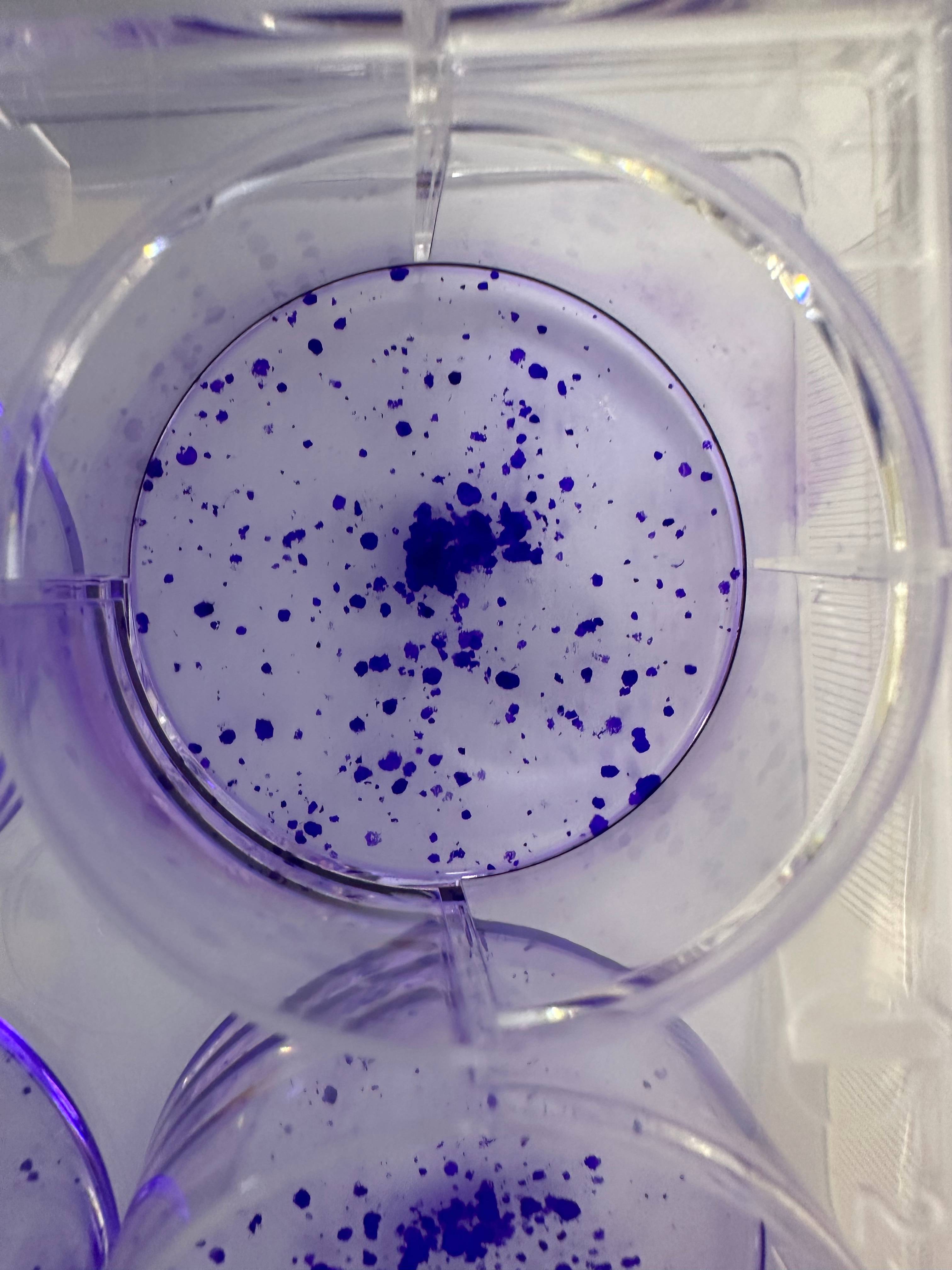

Supplement: Supplementary file 1 [file DataSheet1.zip › Raw image data_Plate cloning/PANC1/si-VTN/V1-2.jpg]

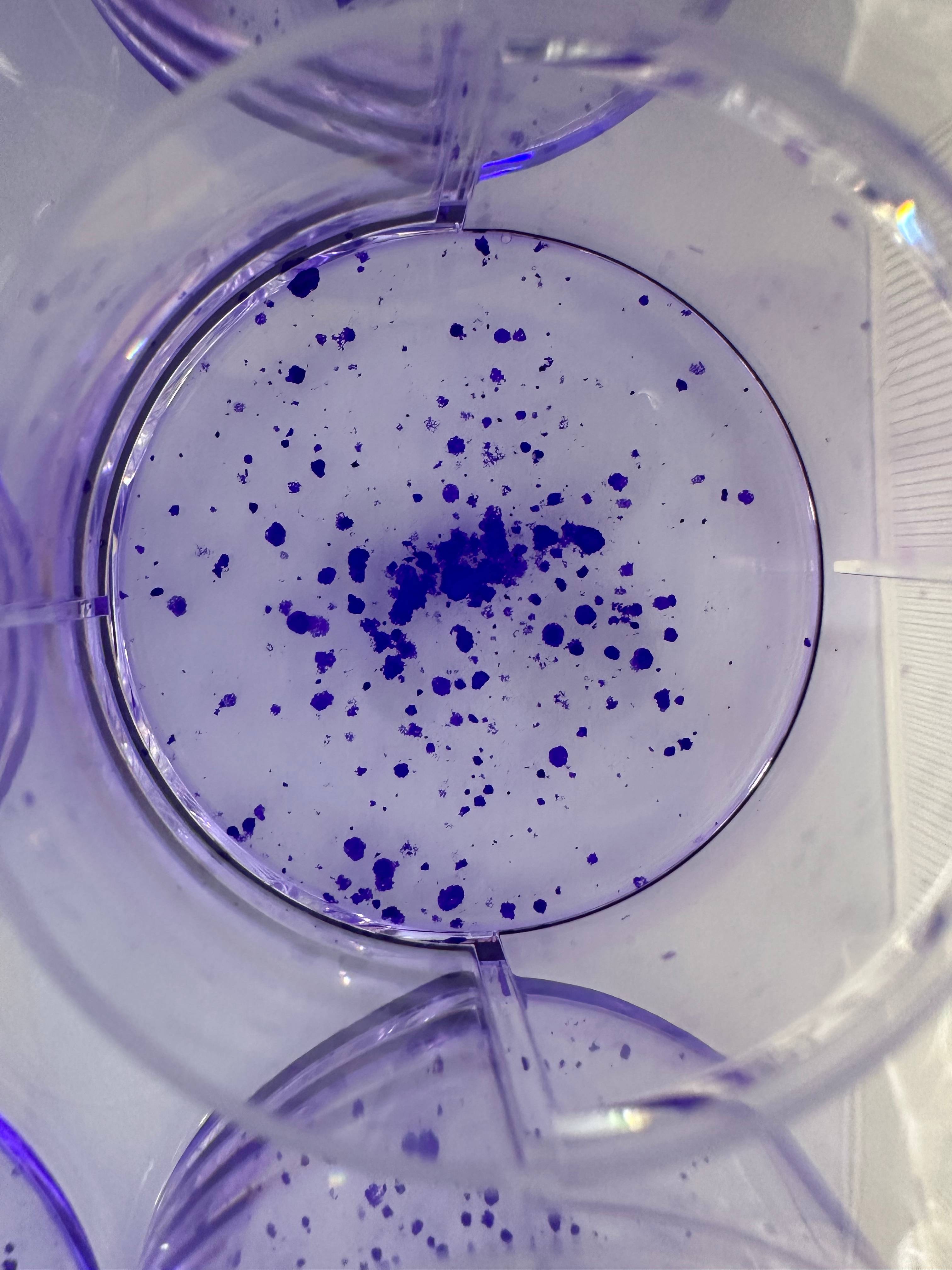

Supplement: Supplementary file 1 [file DataSheet1.zip › Raw image data_Plate cloning/PANC1/si-VTN/V2..jpg]

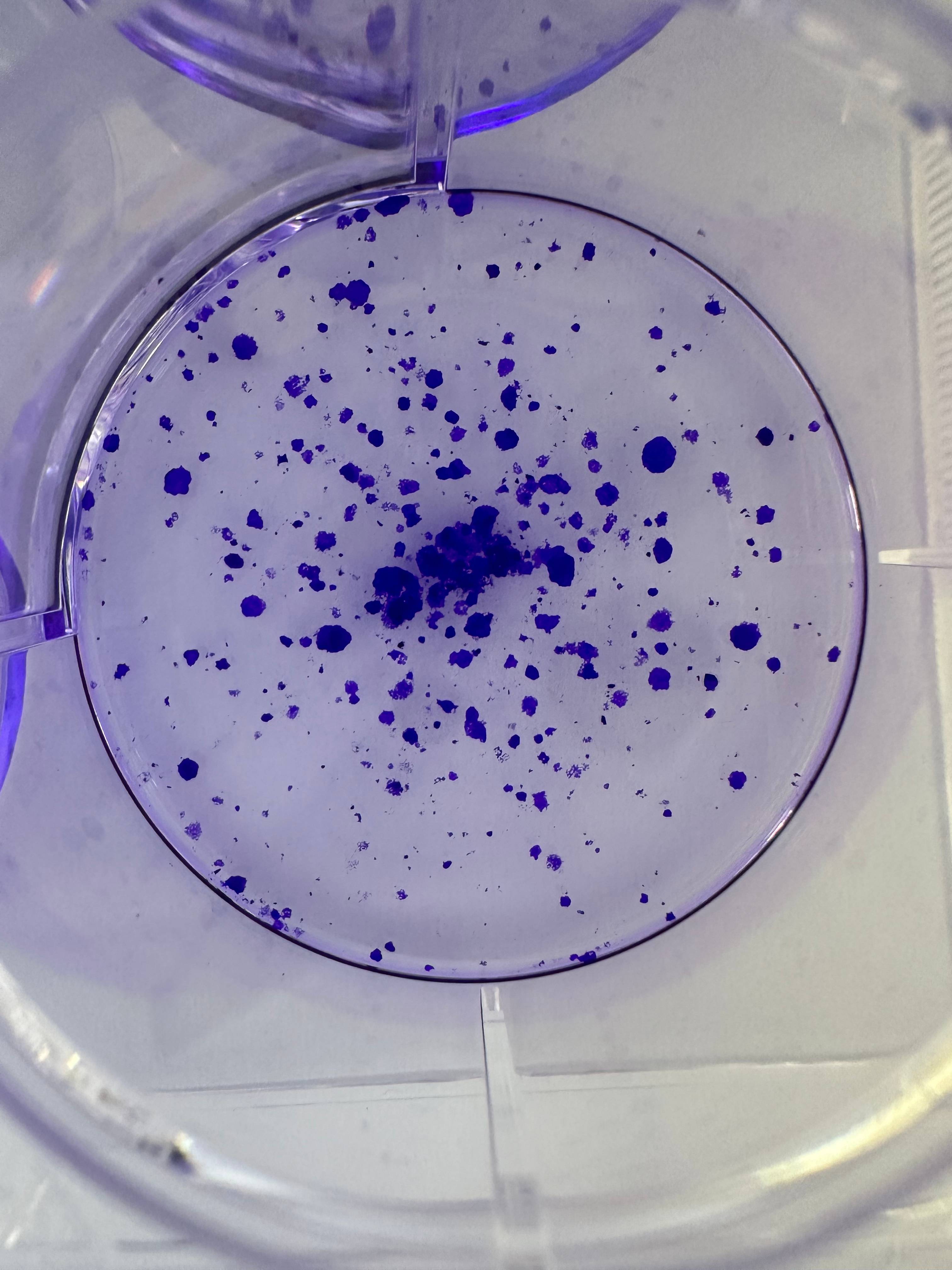

Supplement: Supplementary file 1 [file DataSheet1.zip › Raw image data_Plate cloning/PANC1/si-VTN/V3.jpg]

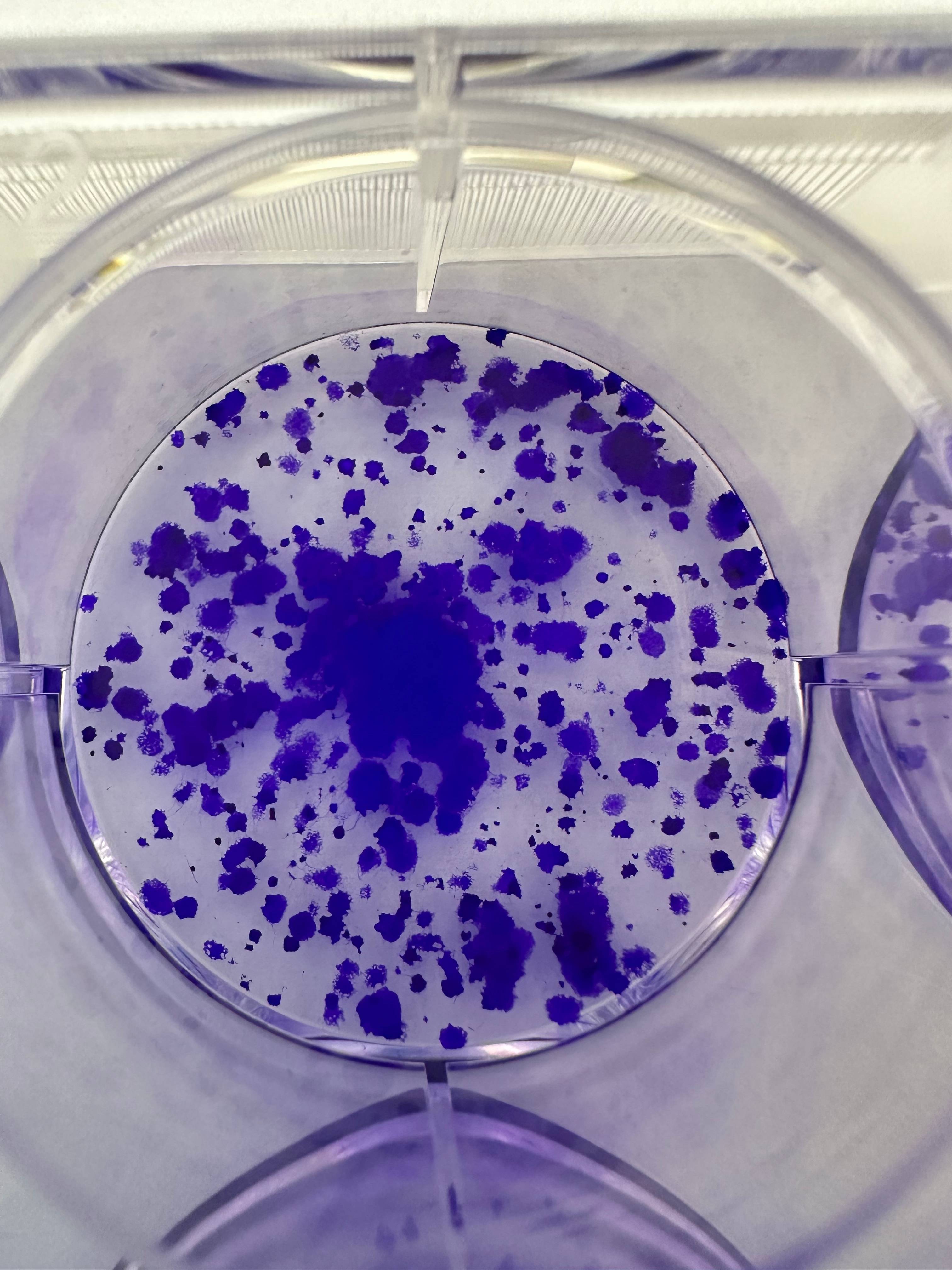

Supplement: Supplementary file 1 [file DataSheet1.zip › Raw image data_Plate cloning/PATU8988/OE-NC/V41-3.jpg]

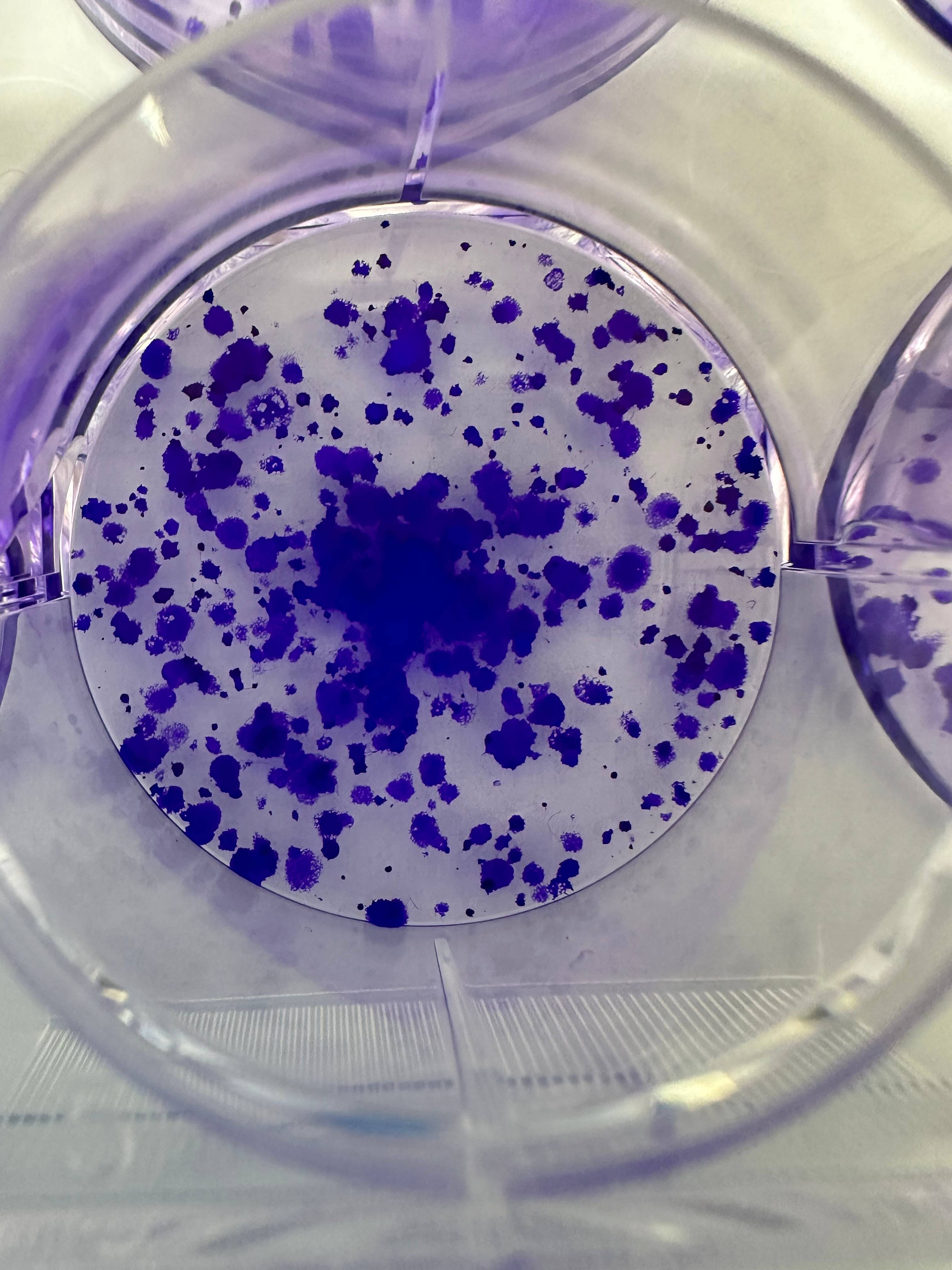

Supplement: Supplementary file 1 [file DataSheet1.zip › Raw image data_Plate cloning/PATU8988/OE-NC/V42.jpg]

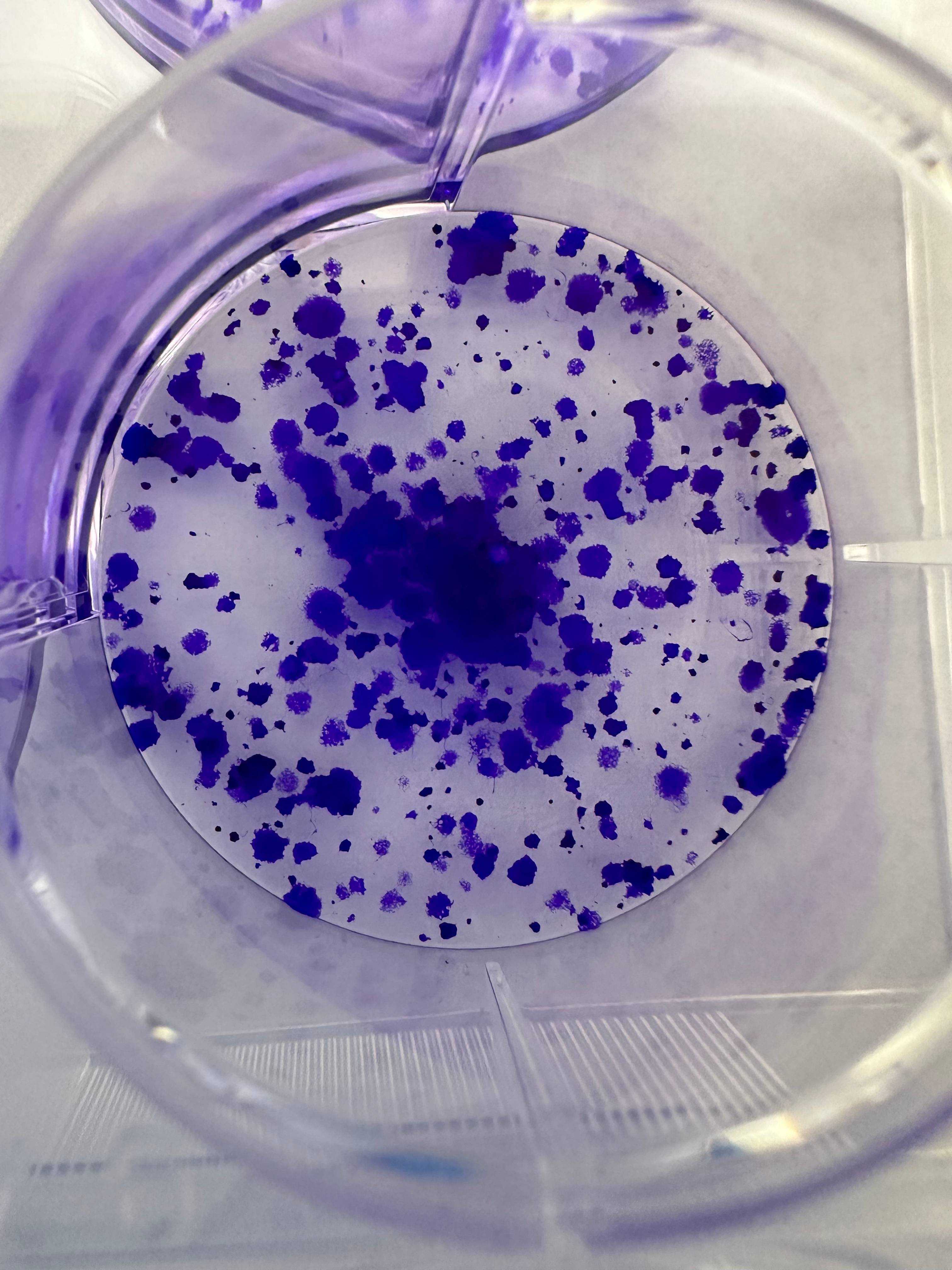

Supplement: Supplementary file 1 [file DataSheet1.zip › Raw image data_Plate cloning/PATU8988/OE-NC/V43.jpg]

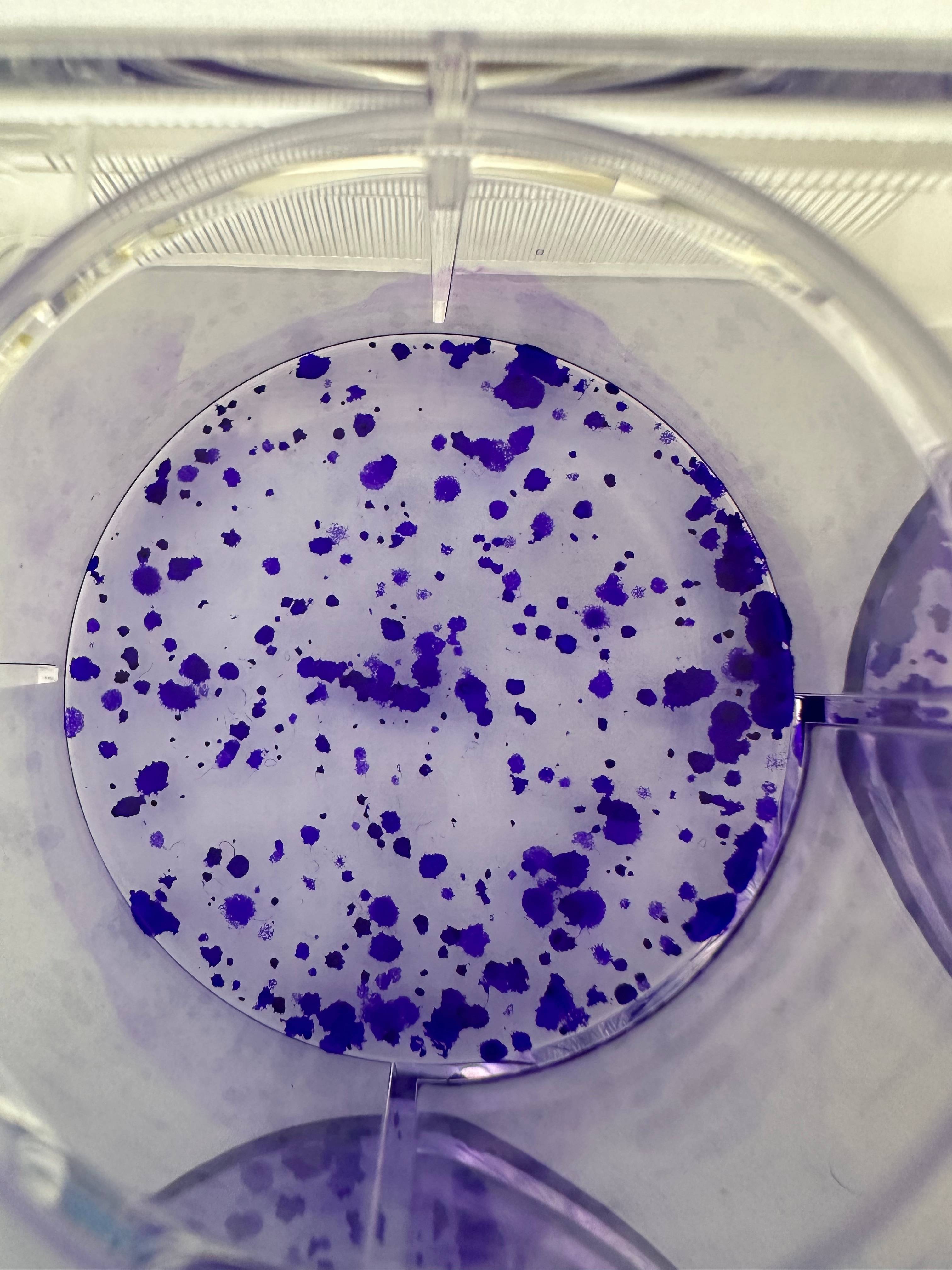

Supplement: Supplementary file 1 [file DataSheet1.zip › Raw image data_Plate cloning/PATU8988/OE-VTN/V31-4.jpg]

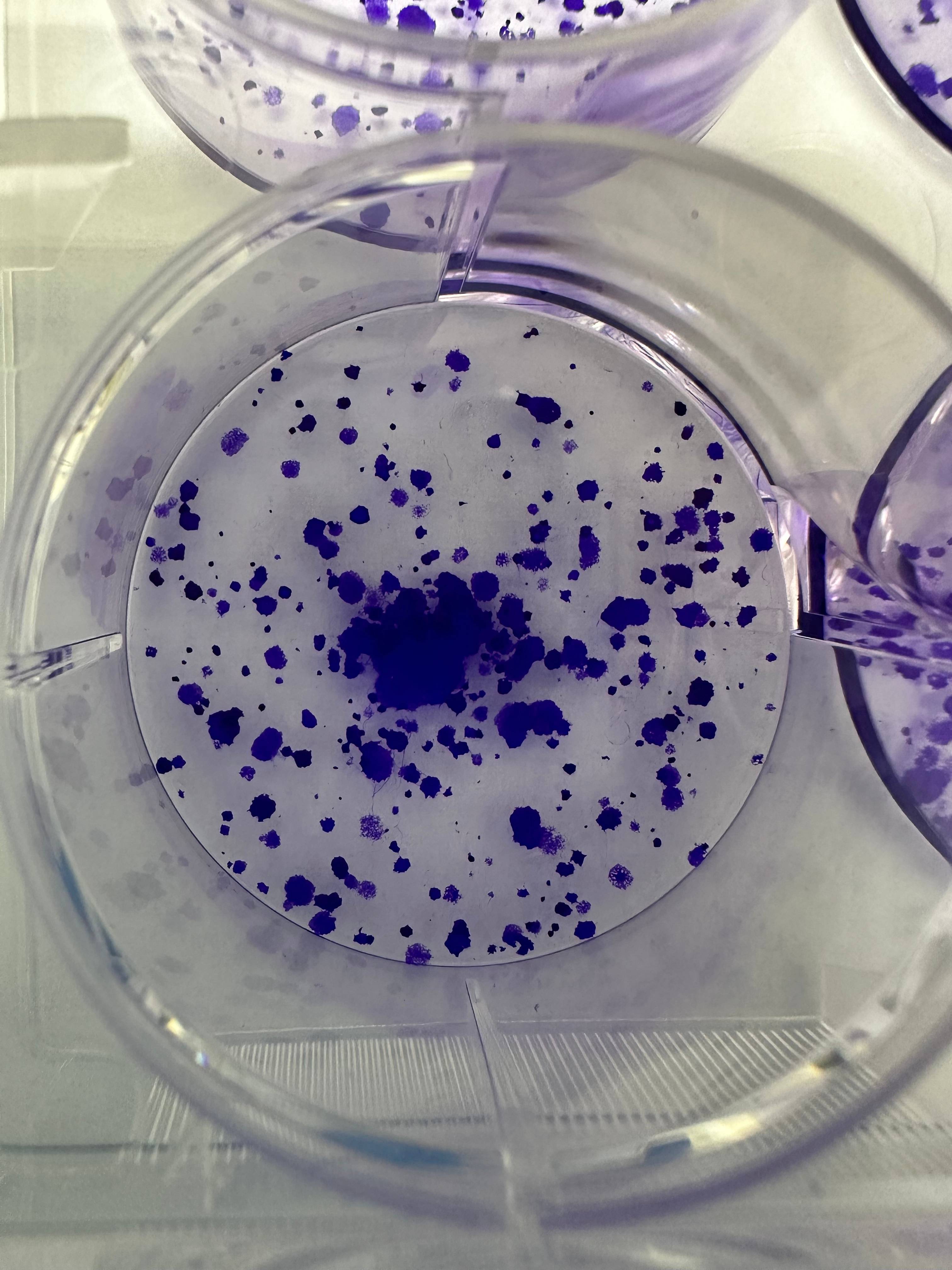

Supplement: Supplementary file 1 [file DataSheet1.zip › Raw image data_Plate cloning/PATU8988/OE-VTN/V32.jpg]

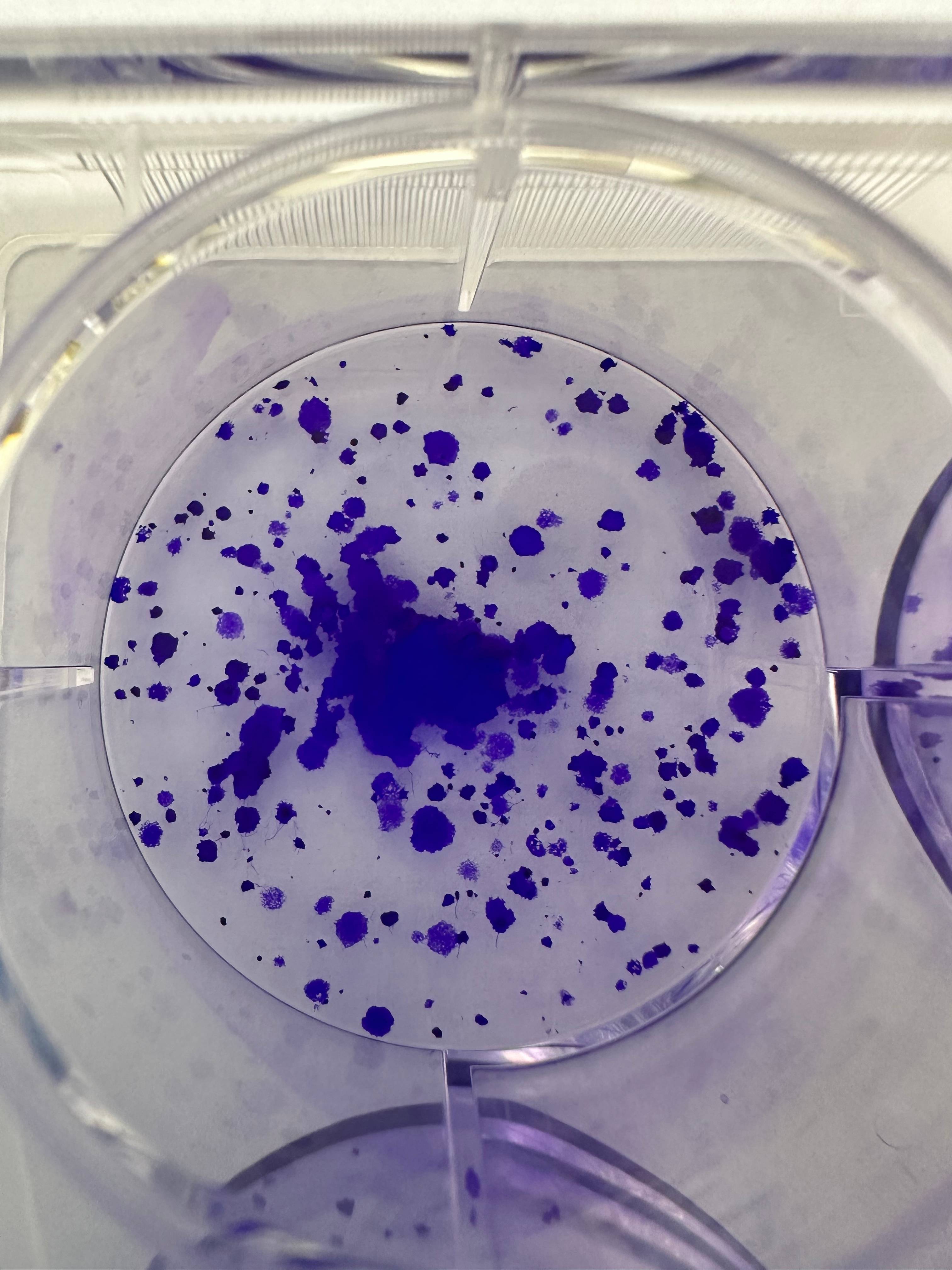

Supplement: Supplementary file 1 [file DataSheet1.zip › Raw image data_Plate cloning/PATU8988/OE-VTN/V33.jpg]

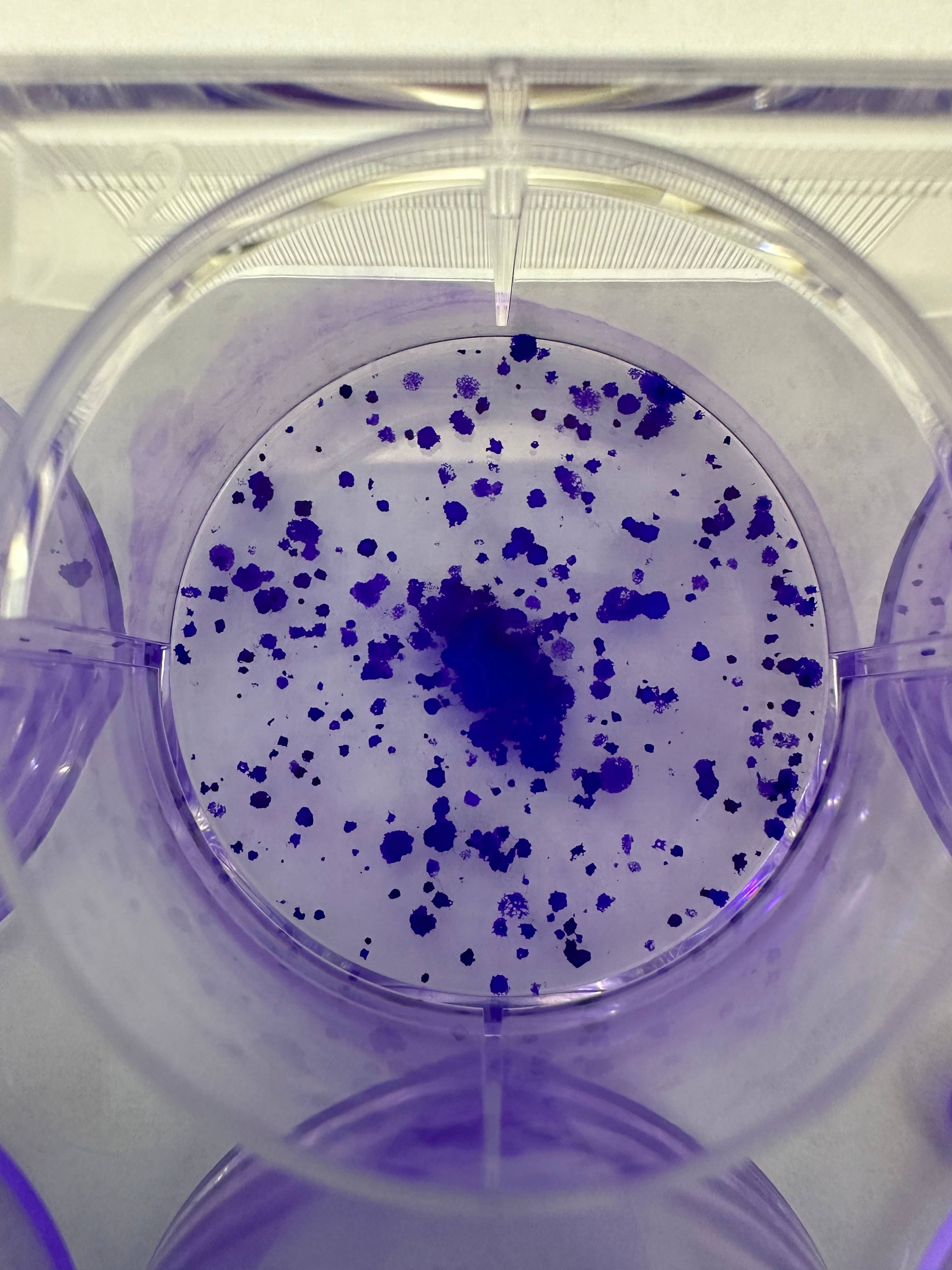

Supplement: Supplementary file 1 [file DataSheet1.zip › Raw image data_Plate cloning/PATU8988/SI-NC/V1.jpg]

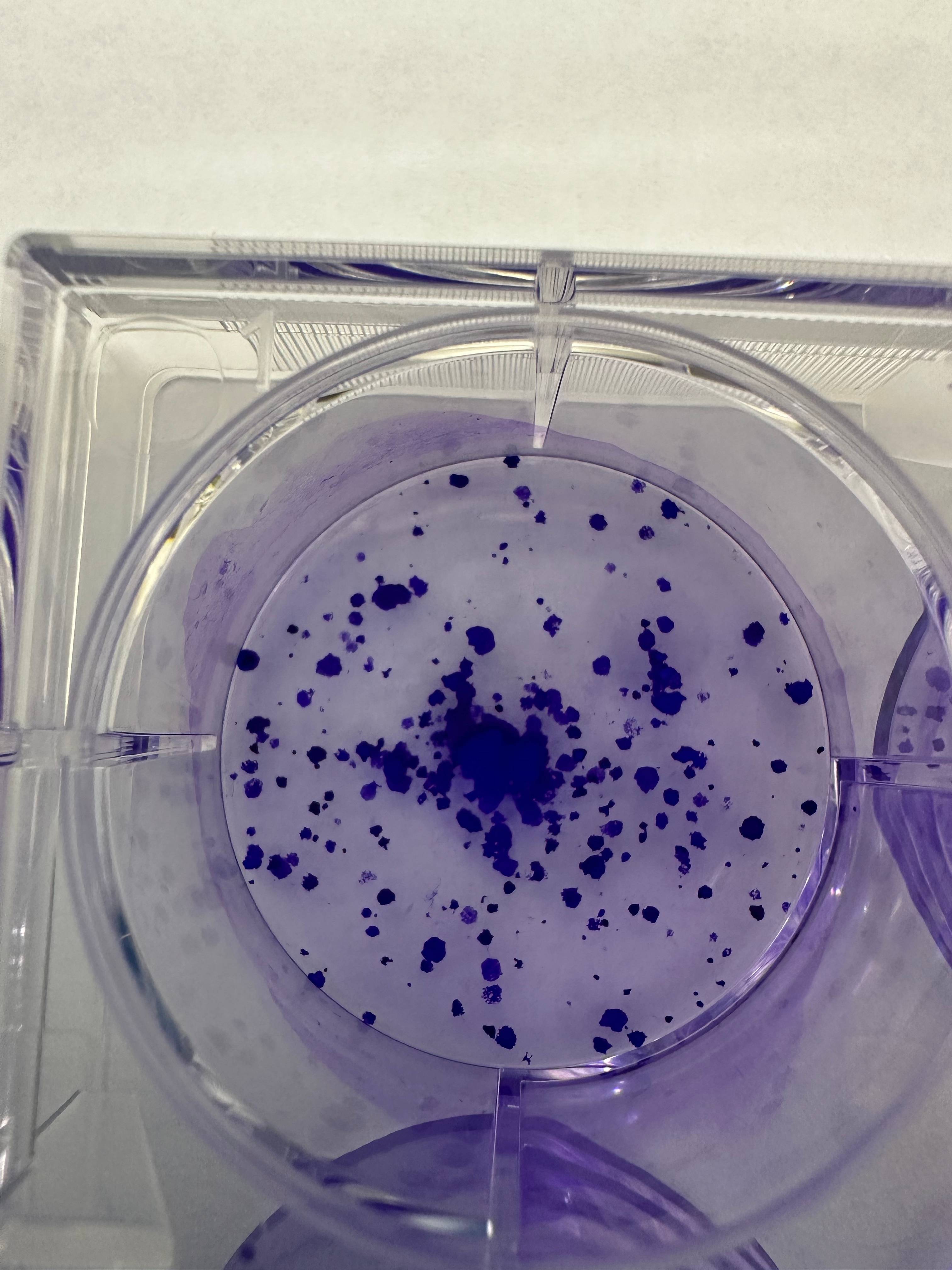

Supplement: Supplementary file 1 [file DataSheet1.zip › Raw image data_Plate cloning/PATU8988/SI-NC/V2.jpg]

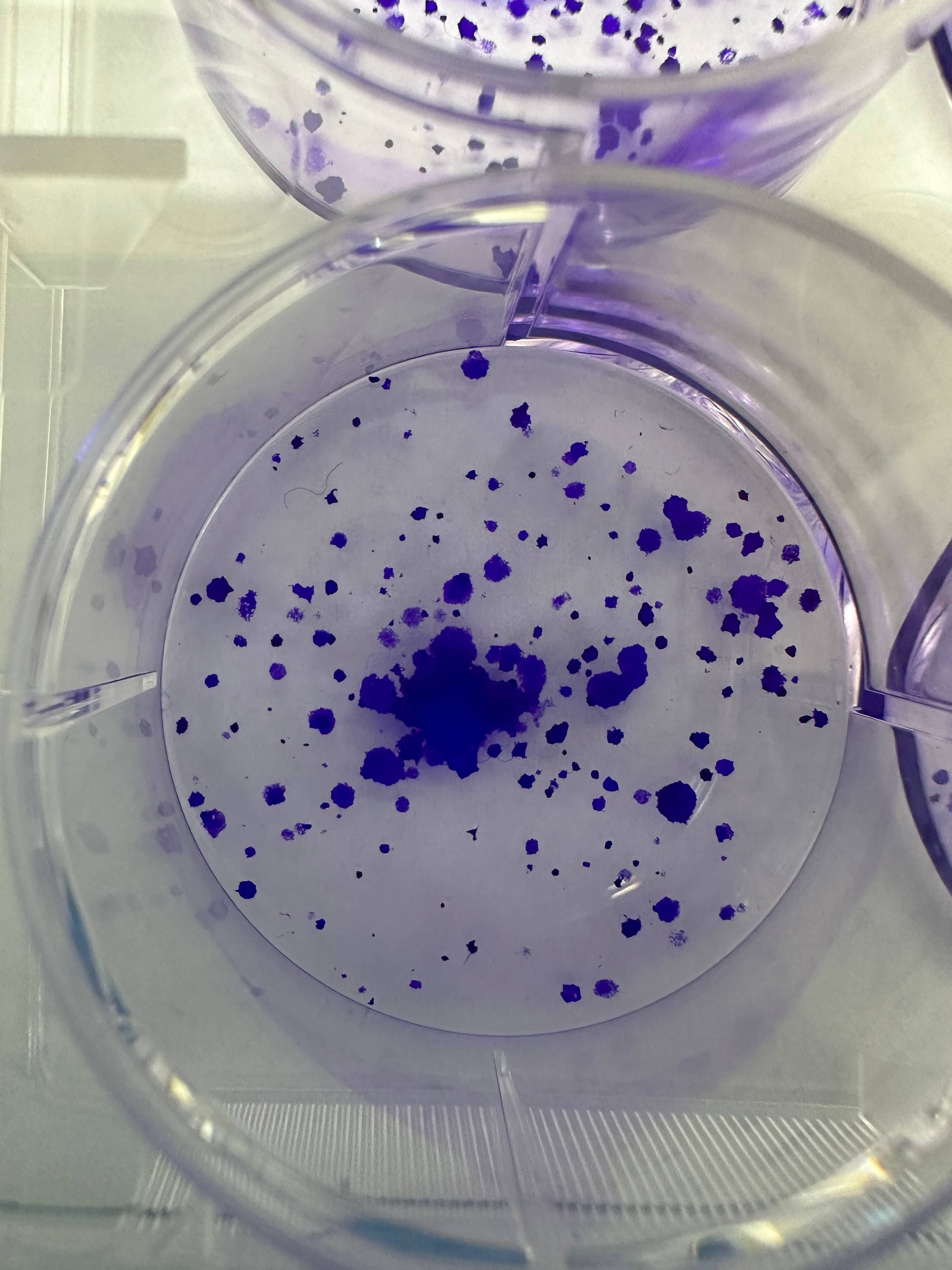

Supplement: Supplementary file 1 [file DataSheet1.zip › Raw image data_Plate cloning/PATU8988/SI-NC/V3-1.jpg]

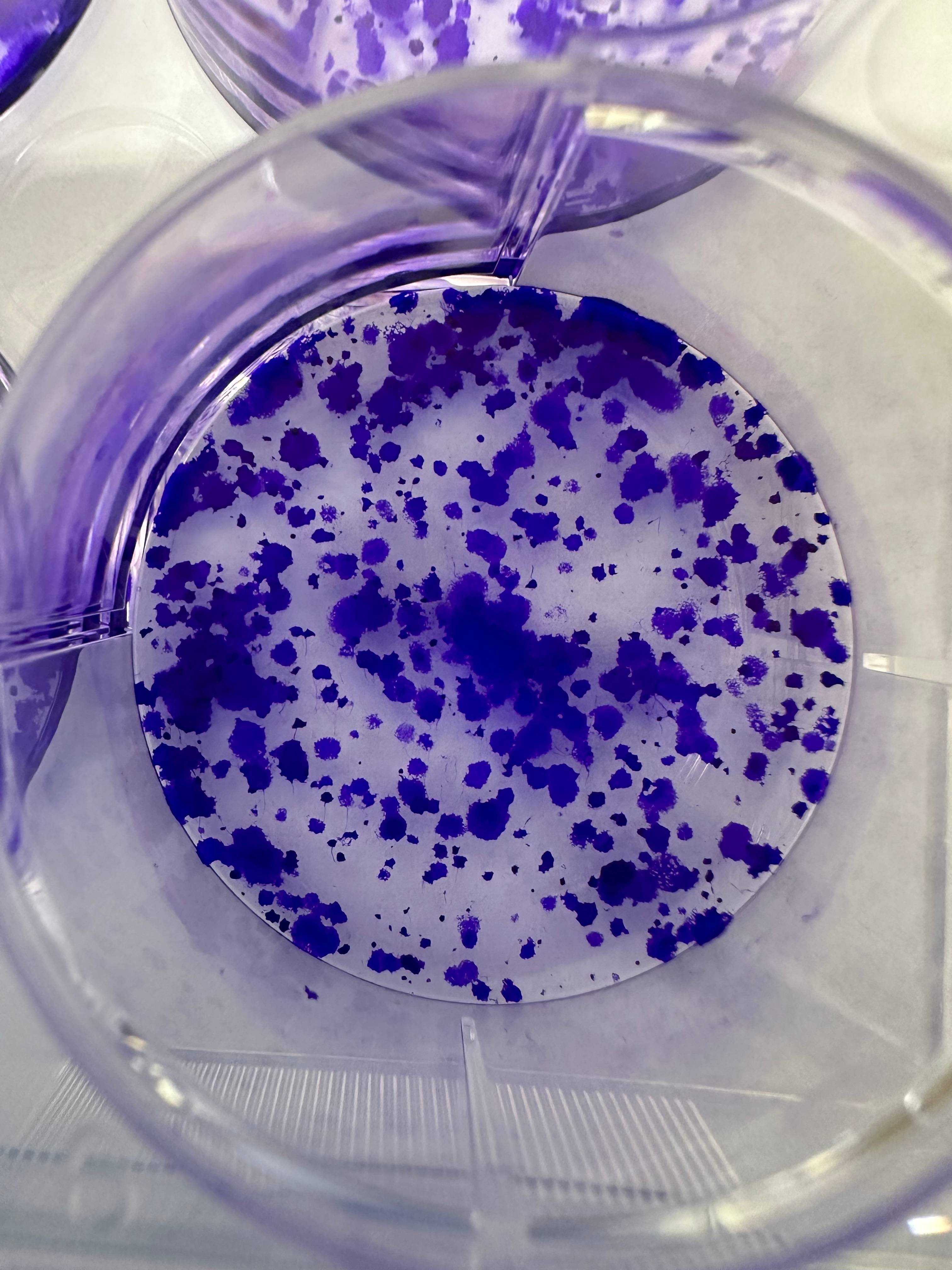

Supplement: Supplementary file 1 [file DataSheet1.zip › Raw image data_Plate cloning/PATU8988/SI-VTN/V21-2.jpg]

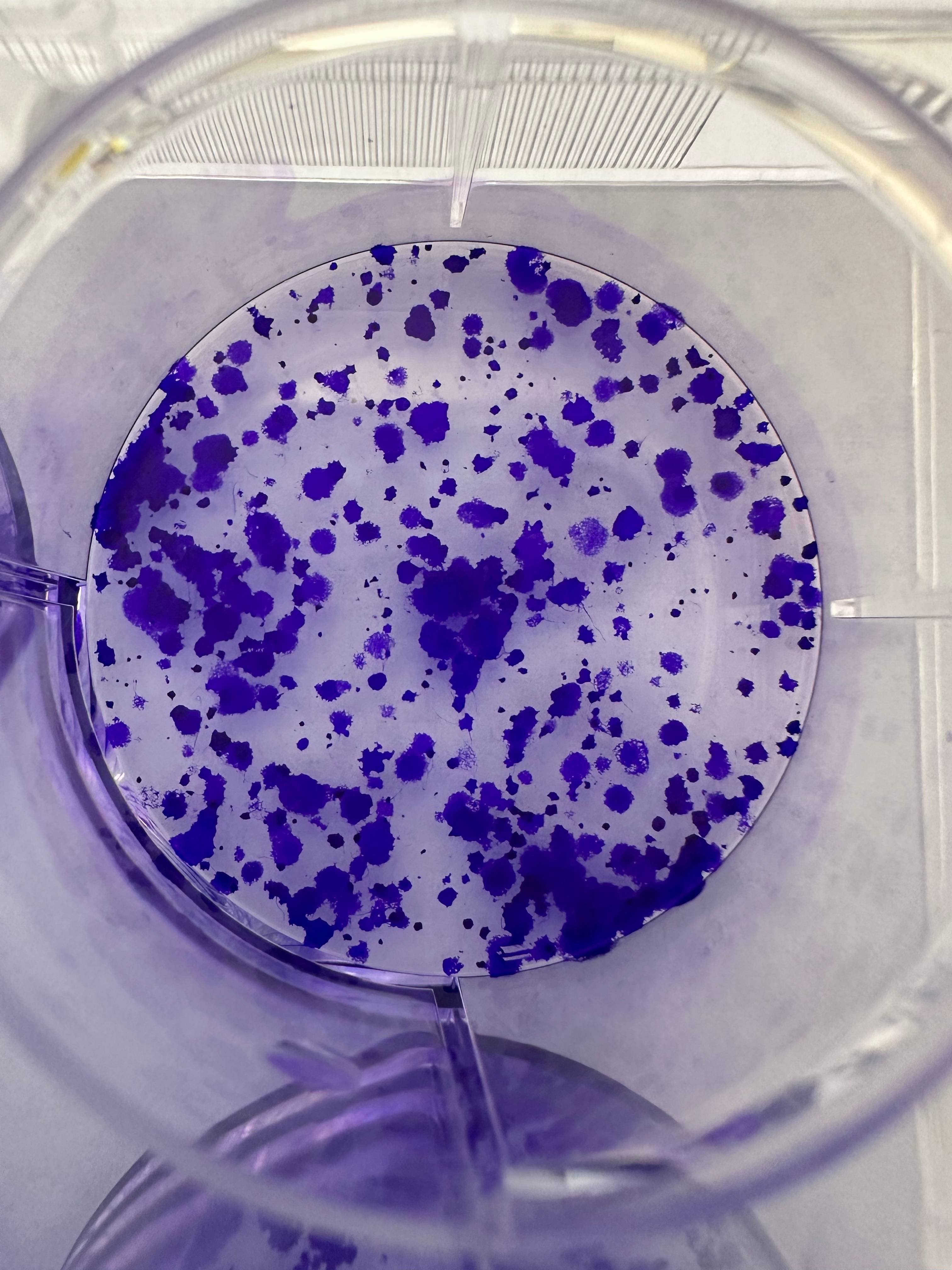

Supplement: Supplementary file 1 [file DataSheet1.zip › Raw image data_Plate cloning/PATU8988/SI-VTN/V22.jpg]

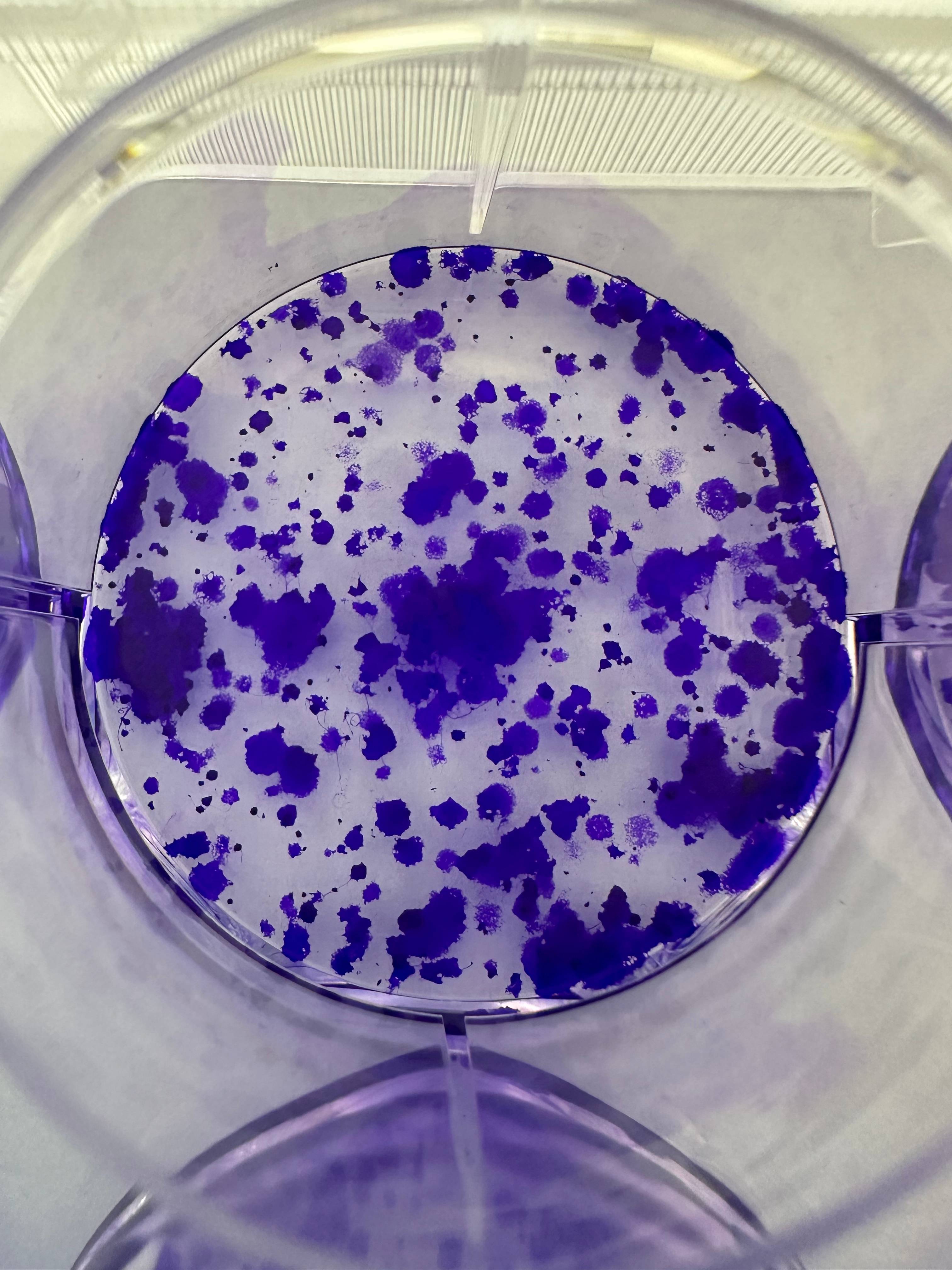

Supplement: Supplementary file 1 [file DataSheet1.zip › Raw image data_Plate cloning/PATU8988/SI-VTN/V23.jpg]

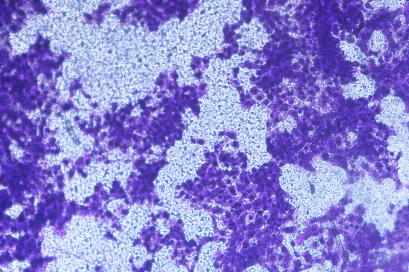

Supplement: Supplementary file 2 [file DataSheet2.zip › Raw image data_Transwell/PANC1/OE-NC-Invasion/V111.png]

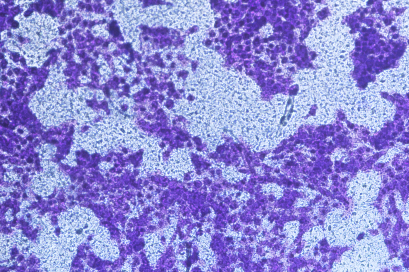

Supplement: Supplementary file 2 [file DataSheet2.zip › Raw image data_Transwell/PANC1/OE-NC-Invasion/V112-5.png]

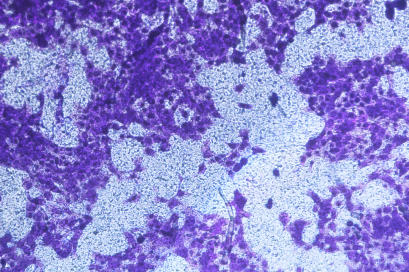

Supplement: Supplementary file 2 [file DataSheet2.zip › Raw image data_Transwell/PANC1/OE-NC-Invasion/V113.png]

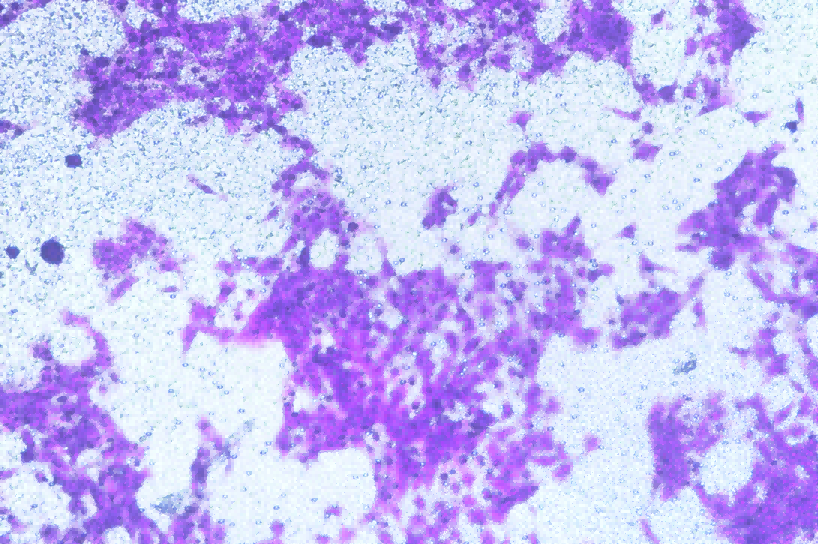

Supplement: Supplementary file 2 [file DataSheet2.zip › Raw image data_Transwell/PANC1/OE-NC-Migration/V151-7.png]

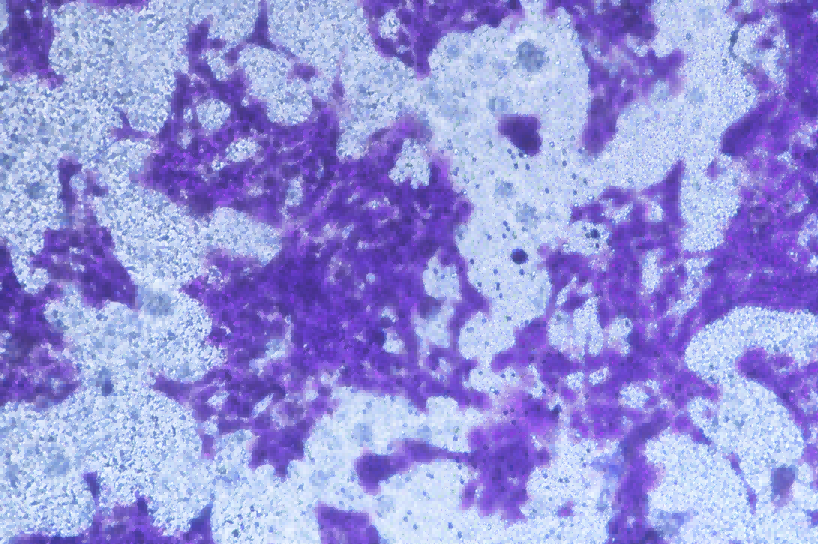

Supplement: Supplementary file 2 [file DataSheet2.zip › Raw image data_Transwell/PANC1/OE-NC-Migration/V152.png]

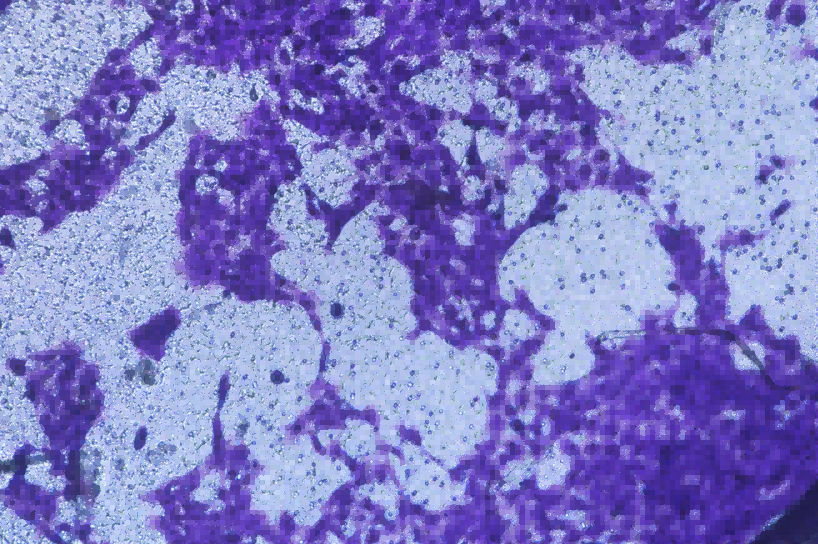

Supplement: Supplementary file 2 [file DataSheet2.zip › Raw image data_Transwell/PANC1/OE-NC-Migration/V153.png]

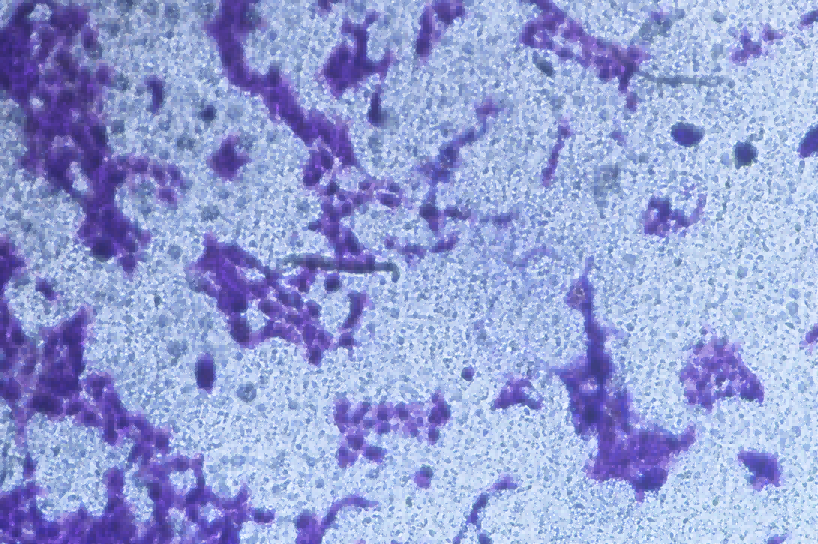

Supplement: Supplementary file 2 [file DataSheet2.zip › Raw image data_Transwell/PANC1/OE-VTN-Invasion/V121-6.png]

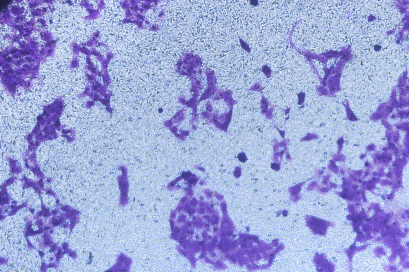

Supplement: Supplementary file 2 [file DataSheet2.zip › Raw image data_Transwell/PANC1/OE-VTN-Invasion/V122.png]

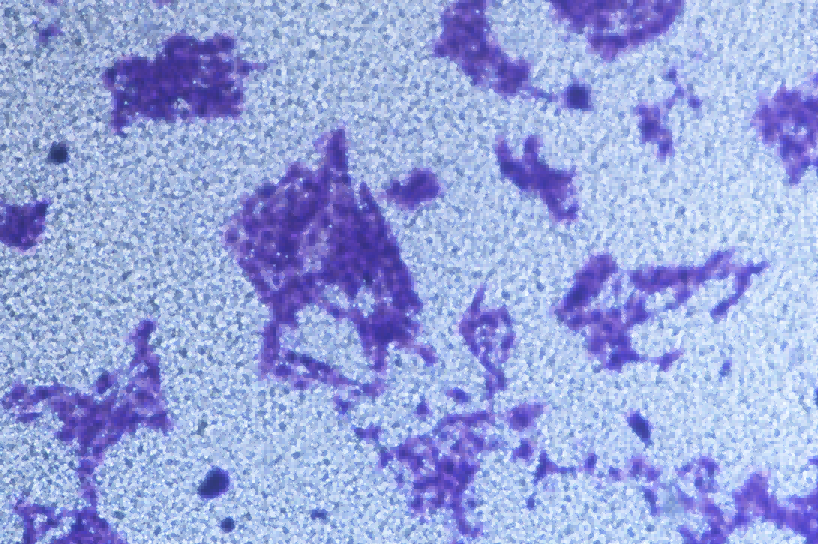

Supplement: Supplementary file 2 [file DataSheet2.zip › Raw image data_Transwell/PANC1/OE-VTN-Invasion/V123.png]

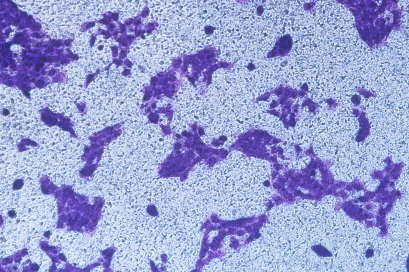

Supplement: Supplementary file 2 [file DataSheet2.zip › Raw image data_Transwell/PANC1/OE-VTN-Migration/V91.png]

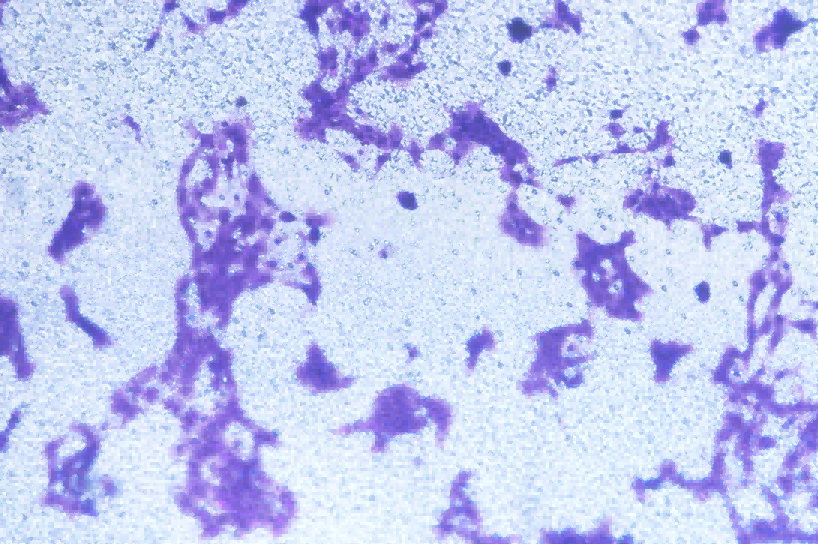

Supplement: Supplementary file 2 [file DataSheet2.zip › Raw image data_Transwell/PANC1/OE-VTN-Migration/V92-8.png]

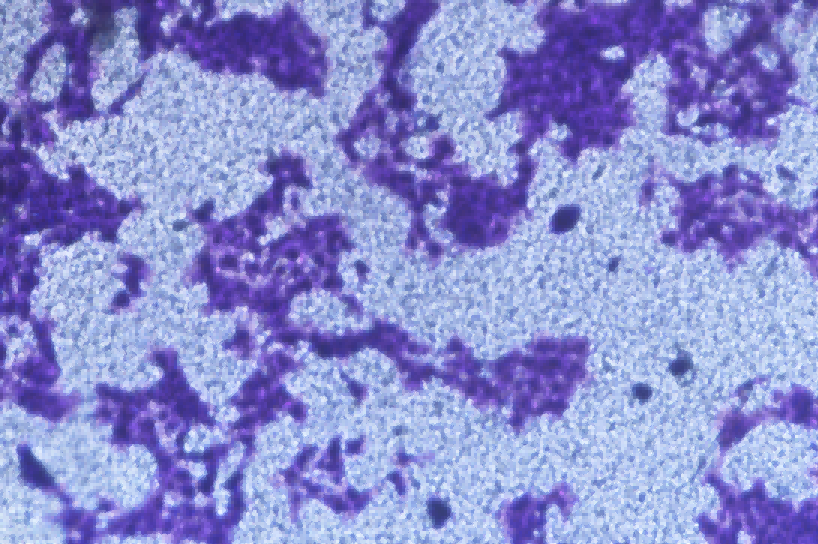

Supplement: Supplementary file 2 [file DataSheet2.zip › Raw image data_Transwell/PANC1/OE-VTN-Migration/V93.png]

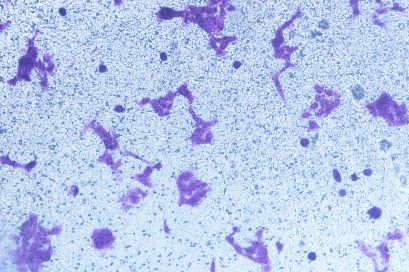

Supplement: Supplementary file 2 [file DataSheet2.zip › Raw image data_Transwell/PANC1/Si-NC-invasion/V61-1.png]

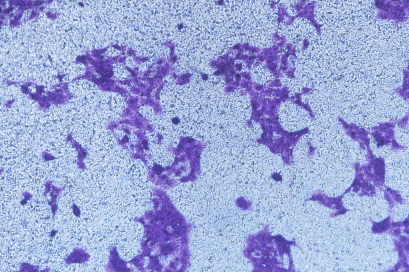

Supplement: Supplementary file 2 [file DataSheet2.zip › Raw image data_Transwell/PANC1/Si-NC-invasion/V62.png]

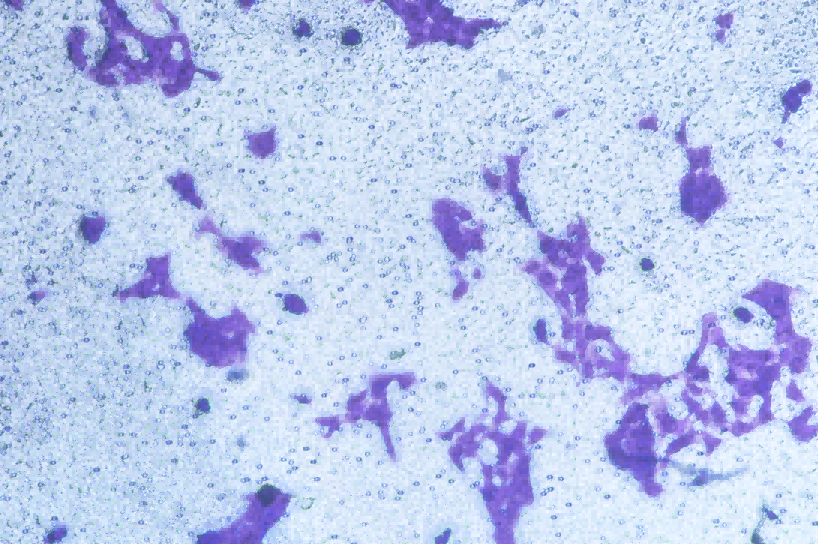

Supplement: Supplementary file 2 [file DataSheet2.zip › Raw image data_Transwell/PANC1/Si-NC-invasion/V63.png]

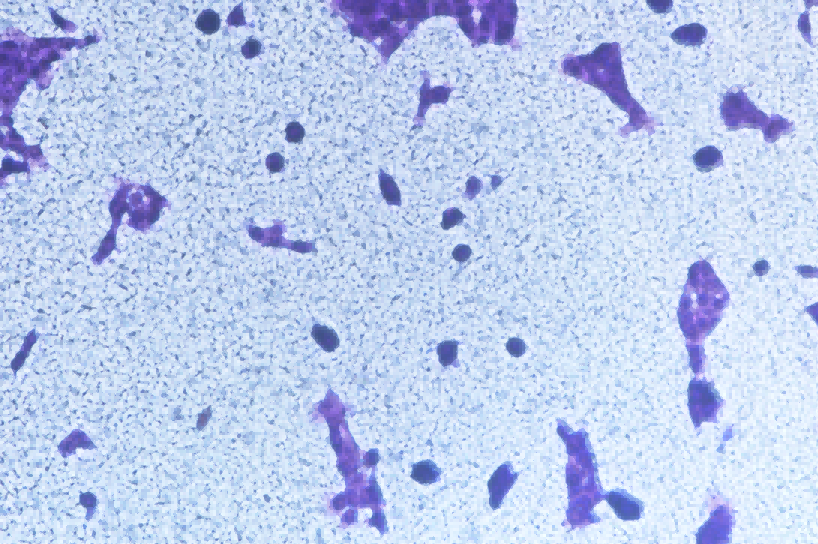

Supplement: Supplementary file 2 [file DataSheet2.zip › Raw image data_Transwell/PANC1/Si-NC-migration/V81-3.png]

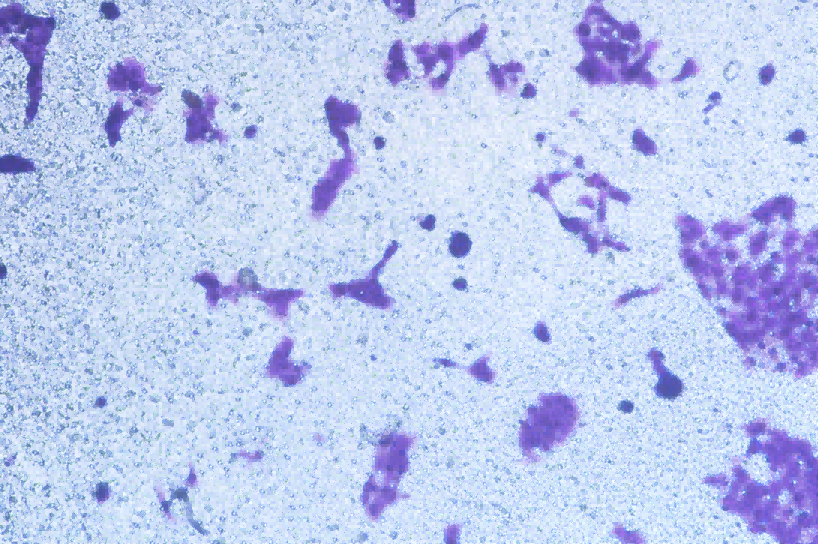

Supplement: Supplementary file 2 [file DataSheet2.zip › Raw image data_Transwell/PANC1/Si-NC-migration/V82.png]

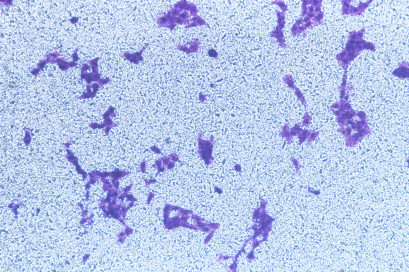

Supplement: Supplementary file 2 [file DataSheet2.zip › Raw image data_Transwell/PANC1/Si-NC-migration/V83.png]

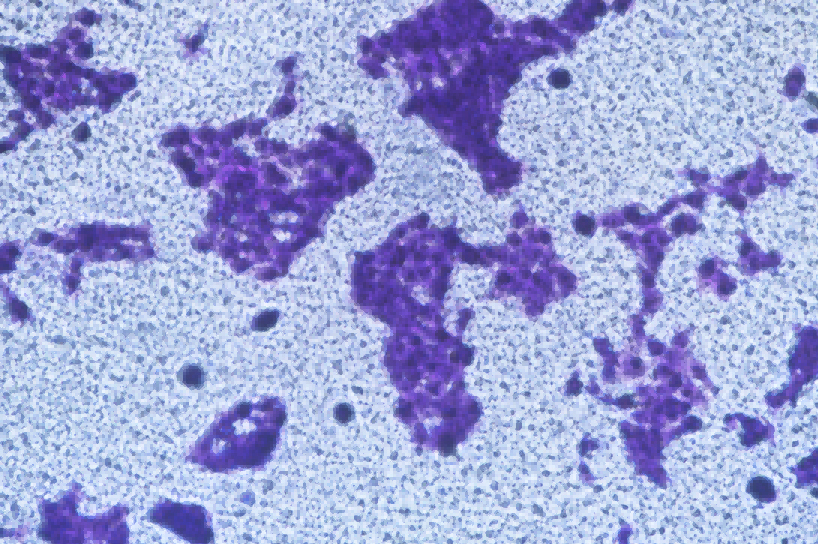

Supplement: Supplementary file 2 [file DataSheet2.zip › Raw image data_Transwell/PANC1/Si-VTN-invasion/V40.png]

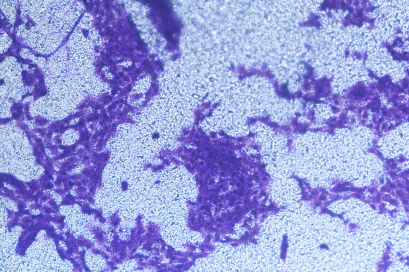

Supplement: Supplementary file 2 [file DataSheet2.zip › Raw image data_Transwell/PANC1/Si-VTN-invasion/V42-2.png]

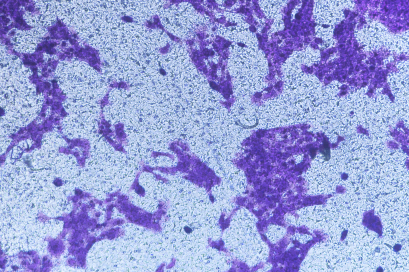

Supplement: Supplementary file 2 [file DataSheet2.zip › Raw image data_Transwell/PANC1/Si-VTN-invasion/V43.png]

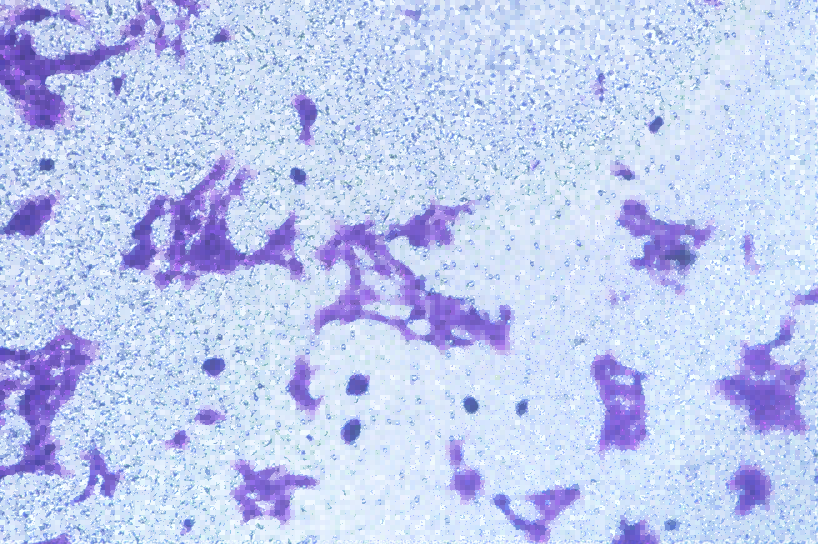

Supplement: Supplementary file 2 [file DataSheet2.zip › Raw image data_Transwell/PANC1/Si-VTN-migration/V101.png]

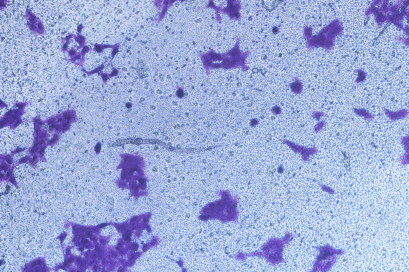

Supplement: Supplementary file 2 [file DataSheet2.zip › Raw image data_Transwell/PANC1/Si-VTN-migration/V102.png]

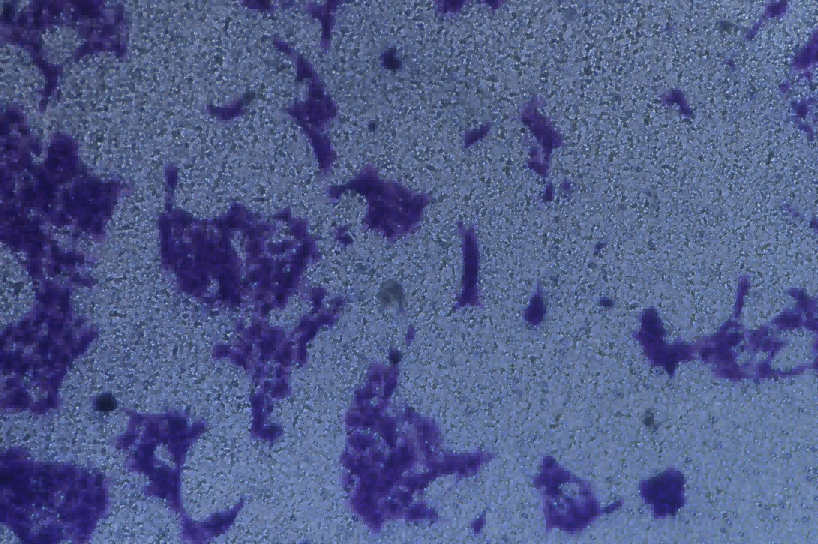

Supplement: Supplementary file 2 [file DataSheet2.zip › Raw image data_Transwell/PANC1/Si-VTN-migration/V103-4.png]

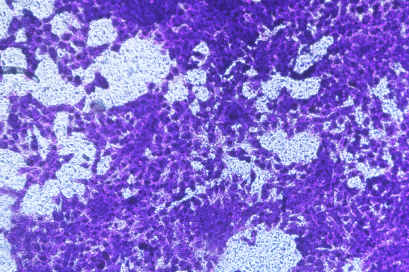

Supplement: Supplementary file 2 [file DataSheet2.zip › Raw image data_Transwell/PATU8988/OE-NC-Invasion/V31-5.png]

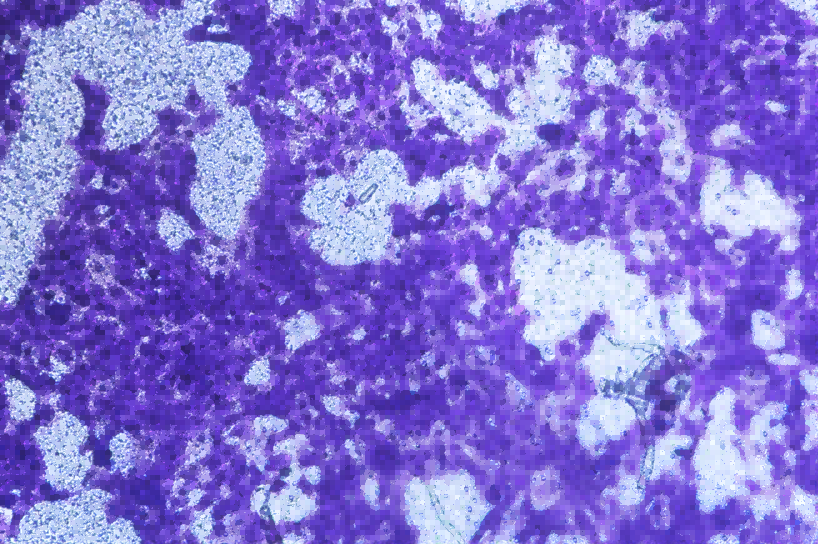

Supplement: Supplementary file 2 [file DataSheet2.zip › Raw image data_Transwell/PATU8988/OE-NC-Invasion/V32.png]

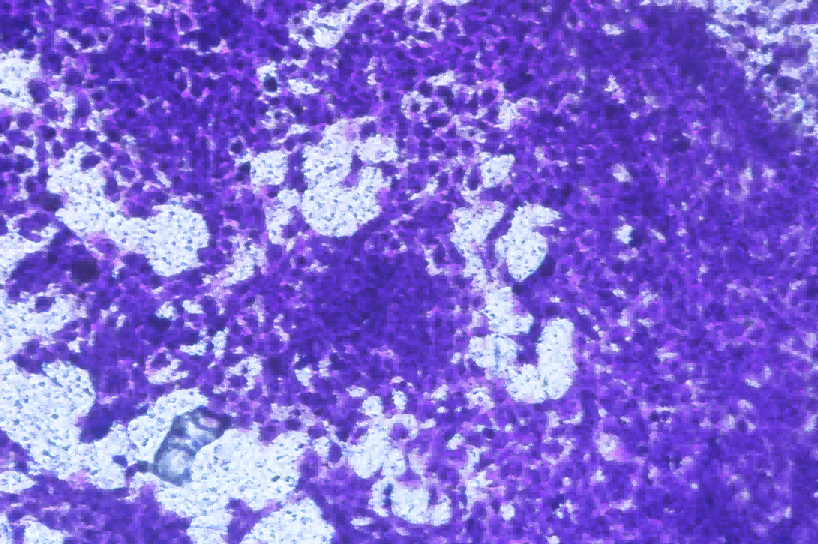

Supplement: Supplementary file 2 [file DataSheet2.zip › Raw image data_Transwell/PATU8988/OE-NC-Invasion/V33.png]

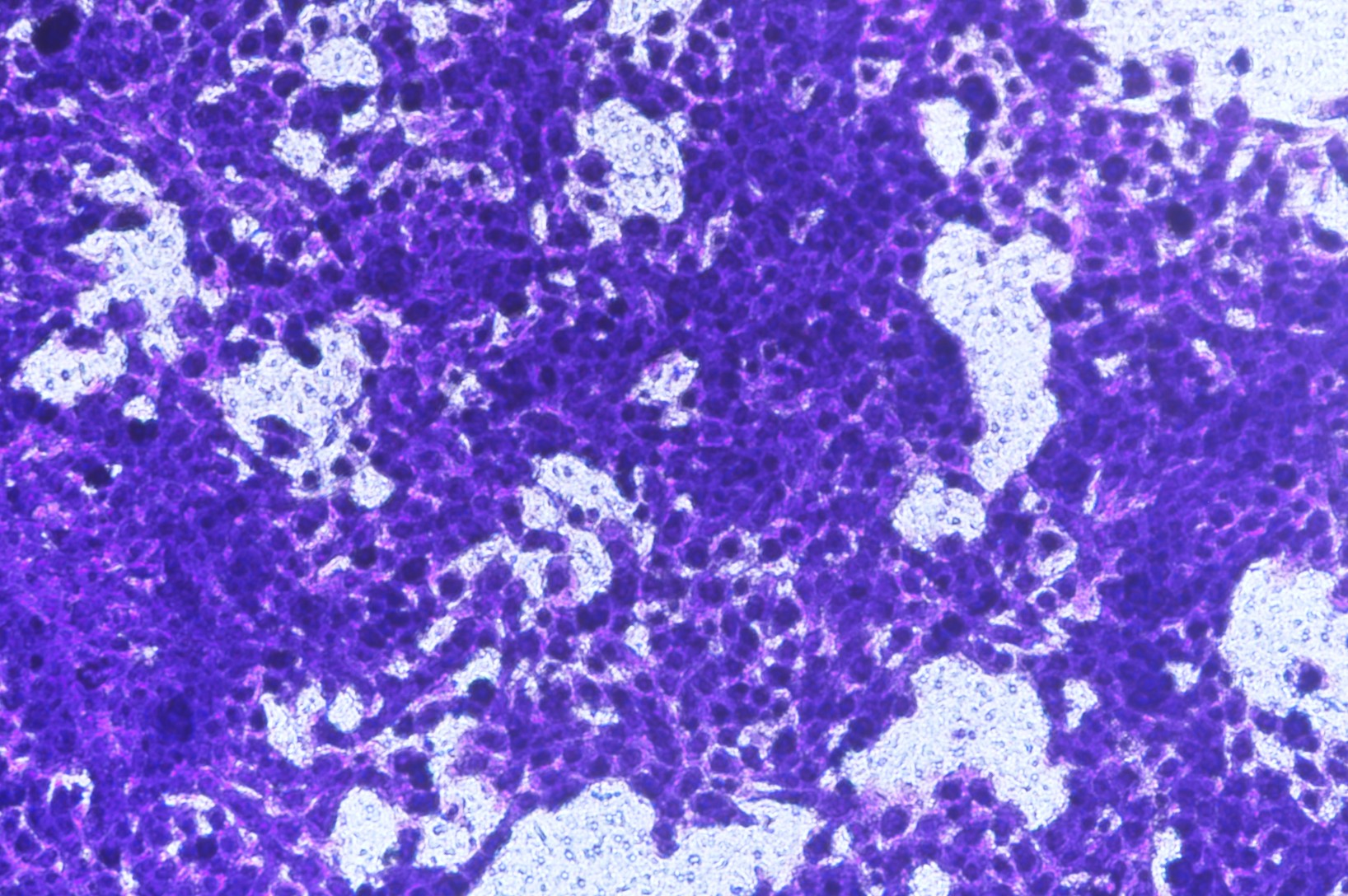

Supplement: Supplementary file 2 [file DataSheet2.zip › Raw image data_Transwell/PATU8988/OE-NC-migration/V.jpg]

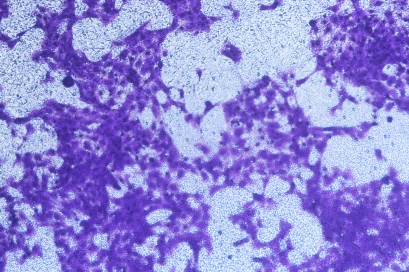

Supplement: Supplementary file 2 [file DataSheet2.zip › Raw image data_Transwell/PATU8988/OE-VTN-Invasion/V1.png]

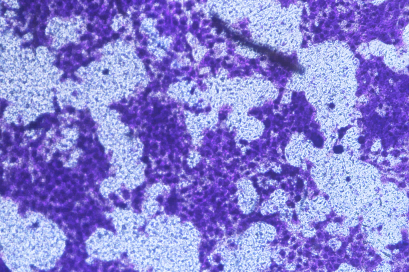

Supplement: Supplementary file 2 [file DataSheet2.zip › Raw image data_Transwell/PATU8988/OE-VTN-Invasion/V2-6.png]

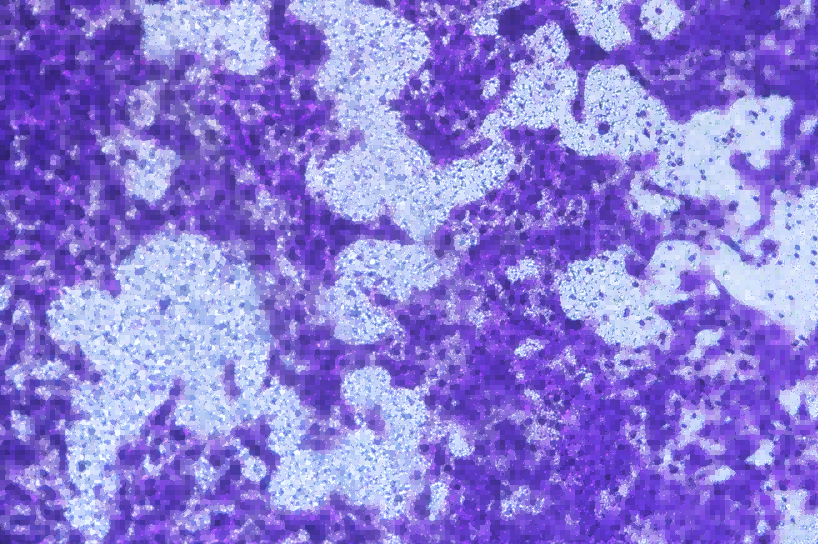

Supplement: Supplementary file 2 [file DataSheet2.zip › Raw image data_Transwell/PATU8988/OE-VTN-Invasion/V3.png]

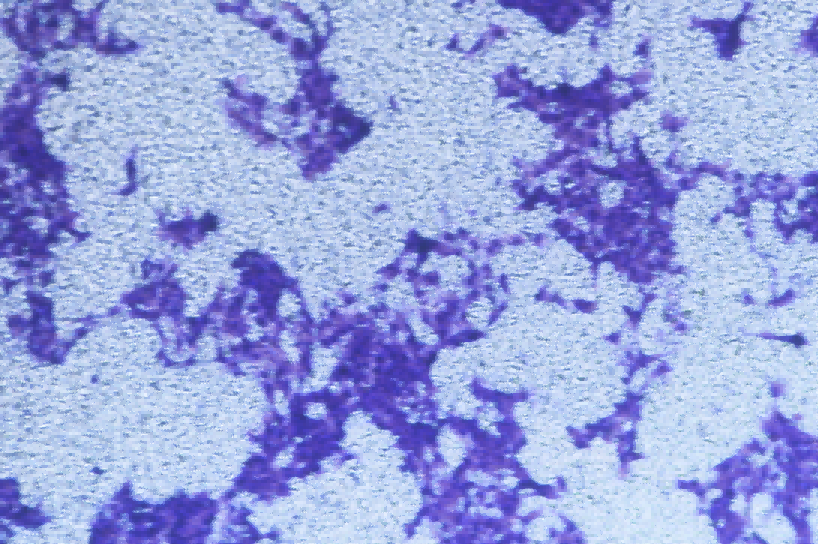

Supplement: Supplementary file 2 [file DataSheet2.zip › Raw image data_Transwell/PATU8988/OE-VTN-migration/V131.png]

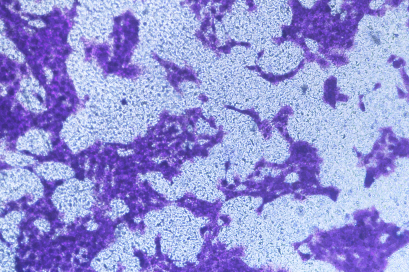

Supplement: Supplementary file 2 [file DataSheet2.zip › Raw image data_Transwell/PATU8988/OE-VTN-migration/V132-8.png]

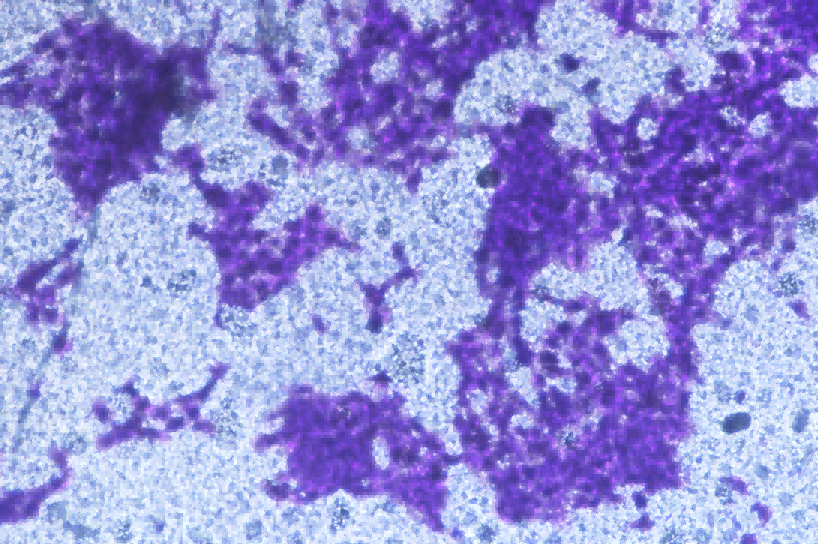

Supplement: Supplementary file 2 [file DataSheet2.zip › Raw image data_Transwell/PATU8988/OE-VTN-migration/V133.png]

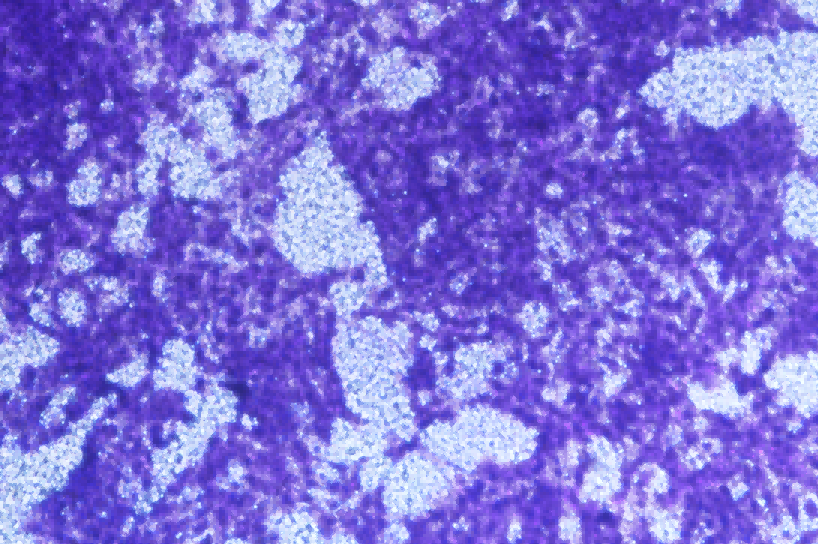

Supplement: Supplementary file 2 [file DataSheet2.zip › Raw image data_Transwell/PATU8988/si-NC-invasion/V141.png]

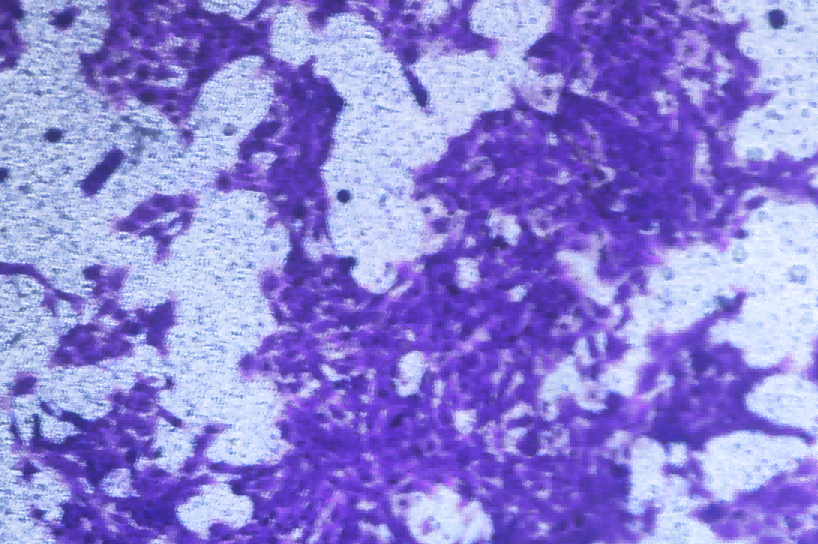

Supplement: Supplementary file 2 [file DataSheet2.zip › Raw image data_Transwell/PATU8988/si-NC-invasion/V142.png]

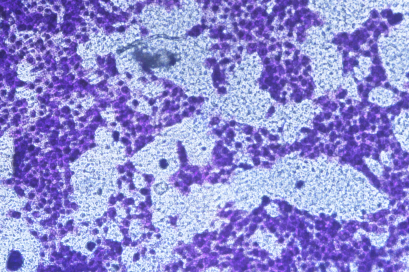

Supplement: Supplementary file 2 [file DataSheet2.zip › Raw image data_Transwell/PATU8988/si-NC-invasion/V143-1.png]

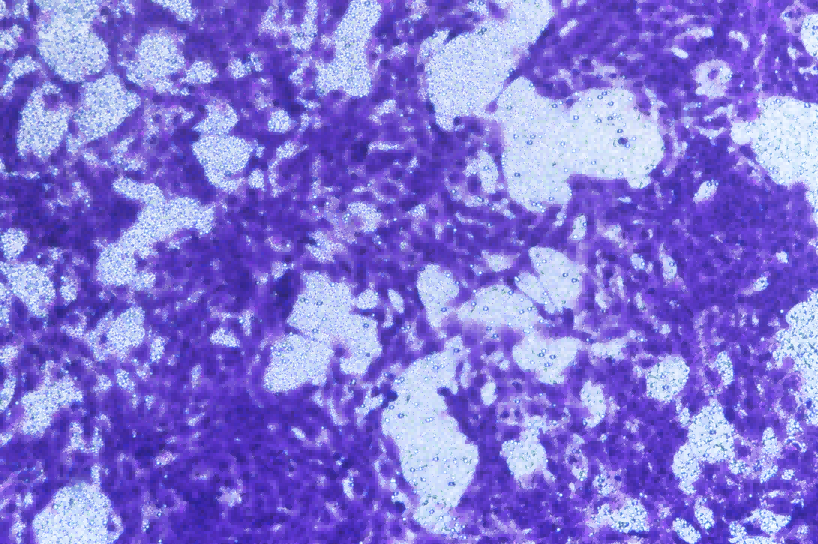

Supplement: Supplementary file 2 [file DataSheet2.zip › Raw image data_Transwell/PATU8988/si-NC-migration/V71-3.png]

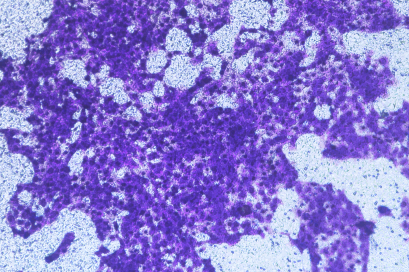

Supplement: Supplementary file 2 [file DataSheet2.zip › Raw image data_Transwell/PATU8988/si-NC-migration/V72.png]

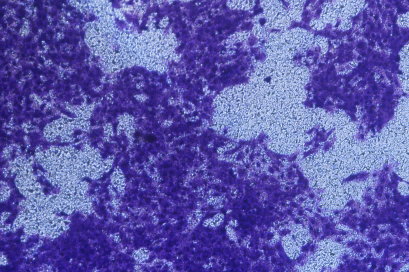

Supplement: Supplementary file 2 [file DataSheet2.zip › Raw image data_Transwell/PATU8988/si-NC-migration/V73.png]

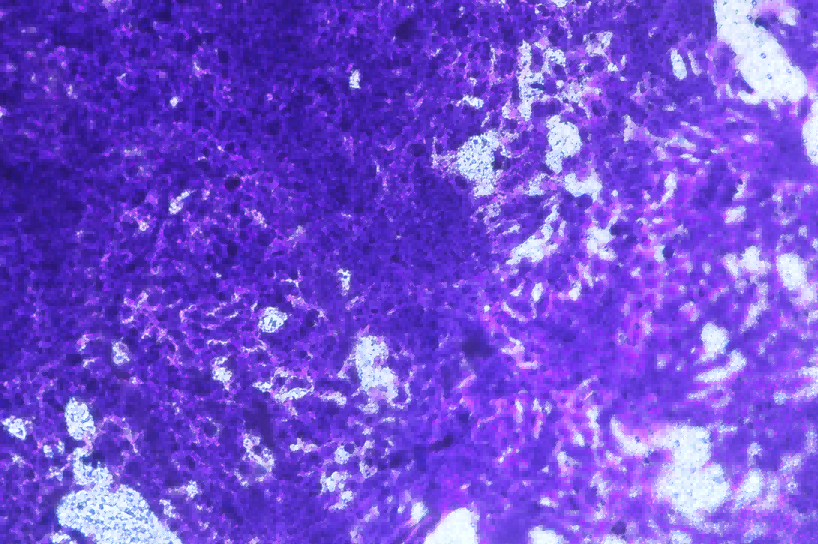

Supplement: Supplementary file 2 [file DataSheet2.zip › Raw image data_Transwell/PATU8988/si-VTN-invasion/V51.png]

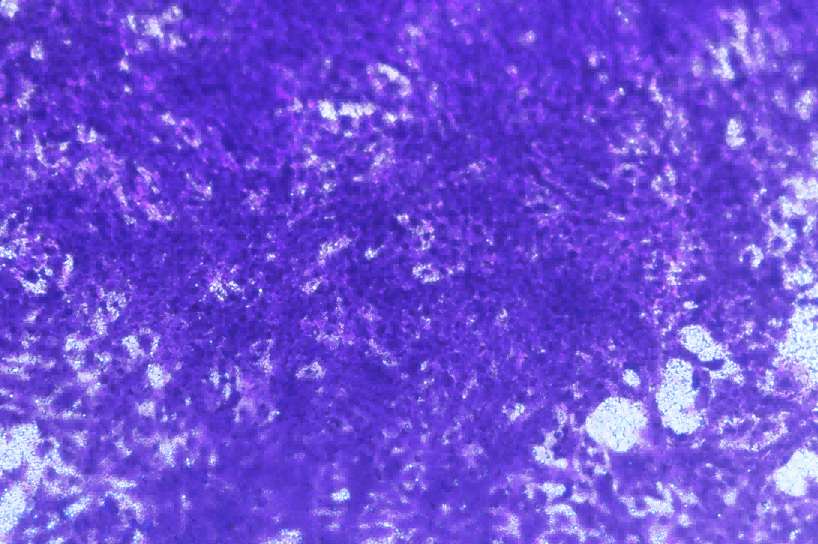

Supplement: Supplementary file 2 [file DataSheet2.zip › Raw image data_Transwell/PATU8988/si-VTN-invasion/V52-2.png]

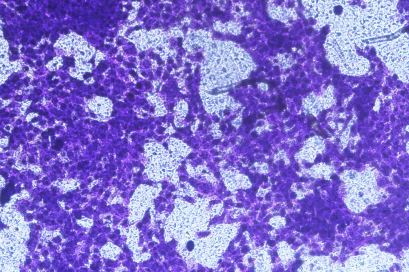

Supplement: Supplementary file 2 [file DataSheet2.zip › Raw image data_Transwell/PATU8988/si-VTN-invasion/V53.png]

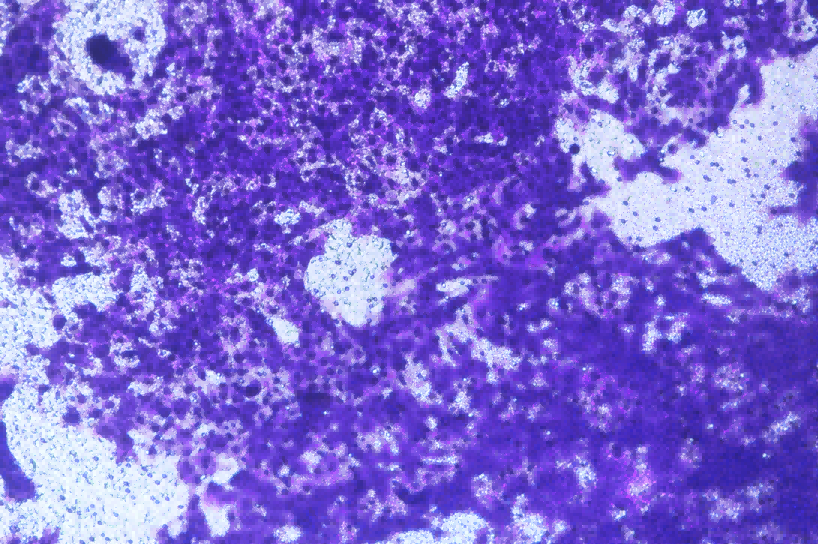

Supplement: Supplementary file 2 [file DataSheet2.zip › Raw image data_Transwell/PATU8988/si-VTN-migration/V21.png]

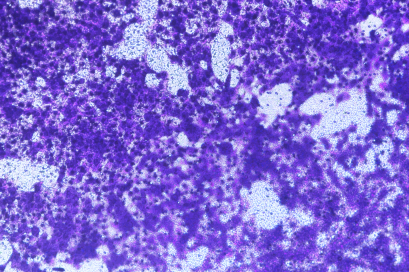

Supplement: Supplementary file 2 [file DataSheet2.zip › Raw image data_Transwell/PATU8988/si-VTN-migration/V22-4.png]

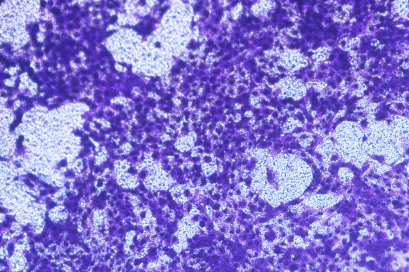

Supplement: Supplementary file 2 [file DataSheet2.zip › Raw image data_Transwell/PATU8988/si-VTN-migration/V23.png]

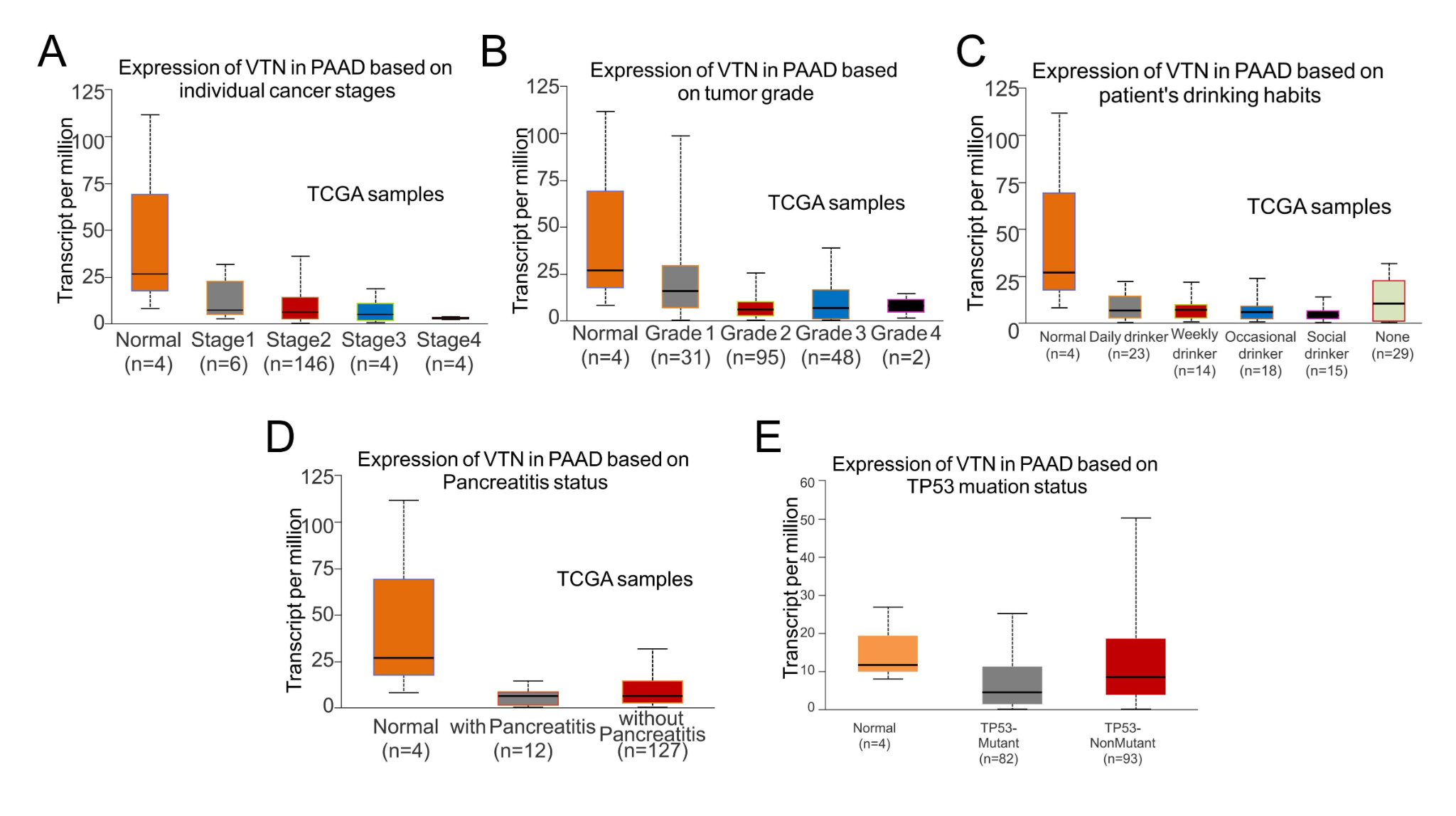

Supplement: Supplementary Figure 1 — Expression profile of VTN in pancreatic cancer. (A) Relationship between VTN expression and tumor stage in pancreatic cancer. (B) Relationship between VTN expression and tumor grade in pancreatic cancer. (C) Relationship between VTN expression and alcohol consumption in pancreatic cancer. (D) Relationship between VTN expression and history of chronic pancreatitis in pancreatic cancer. (E) Relationship between VTN expression and TP53 mutant in pancreatic cancer. [file Image1.jpeg]

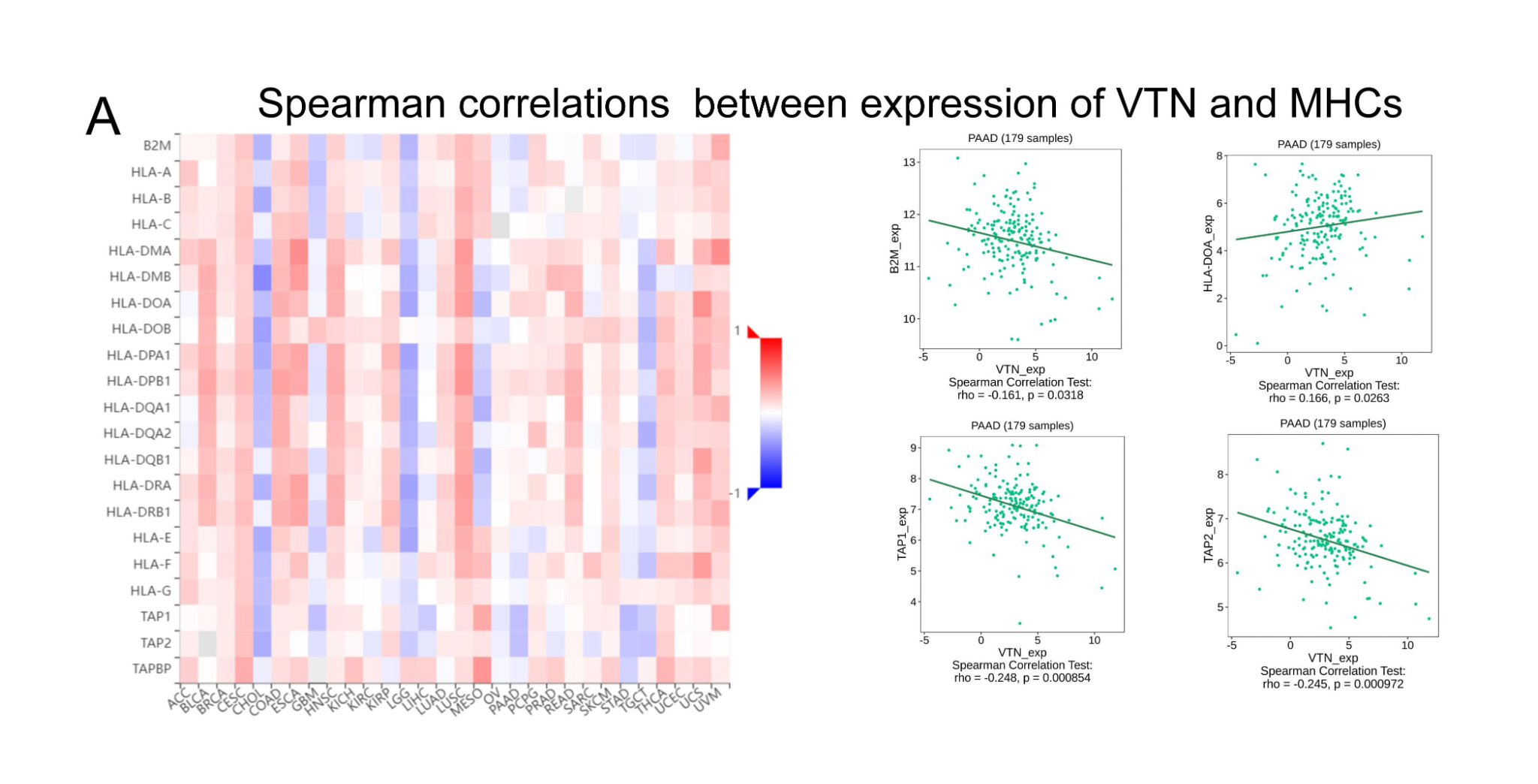

Supplement: Supplementary Figure 2 — The expression of VTN was correlated with MHCs. [file Image2.jpeg]
